# Transcriptomic and Functional Evidence That miRNA193a-3p Inhibits Lymphatic Endothelial Cell (LEC) and LEC + MCF-7 Spheroid Growth Directly and by Altering MCF-7 Secretome

**DOI:** 10.3390/cells12030389

**Published:** 2023-01-21

**Authors:** Giovanna Azzarito, Margit Henry, Tamara Rotshteyn, Brigitte Leeners, Raghvendra K. Dubey

**Affiliations:** 1 Department of Reproductive Endocrinology, University Hospital Zurich, 8952 Schlieren, Switzerland; 2 Center for Physiology, Faculty of Medicine and University Hospital Cologne, University of Cologne, 50931 Cologne, Germany; 3 Institute of Neurophysiology and Center for Molecular Medicine Cologne (CMMC), University of Cologne, 50931 Cologne, Germany; 4 Department of Pharmacology & Chemical Biology, University of Pittsburgh, Pittsburgh, PA 15219, USA

**Keywords:** lymphatic endothelial cells, MCF-7 cells, miRNA, secretome, conditioned medium, breast cancer, proliferation, transcriptome, DRGs, spheroids

## Abstract

MicroRNA 193a-3p (miR193a-3p) is a short non-coding RNA with tumor suppressor properties. Breast cancer (BC) progression is governed by active interaction between breast cancer cells, vascular (V)/lymphatic (L) endothelial cells (ECs), and BC secretome. We have recently shown that miR193a-3p, a tumor suppressor miRNA, inhibits MCF-7 BC cell-driven growth of VECs via direct antimitogenic actions and alters MCF-7 secretome. Since LEC-BC cross-talk plays a key role in BC progression, we investigated the effects of miR193a-3p on MCF-7 secretome and estradiol-mediated growth effects in LECs and LEC + MCF-7 spheroids, and delineated the underlying mechanisms. Transfection of LECs with miR193a-3p, as well as secretome from MCF-7 transfected cells, inhibited LEC growth, and these effects were mimicked in LEC + MCF-7 spheroids. Moreover, miR193a-3p inhibited ERK1/2 and Akt phosphorylation in LECs and LEC + MCF-7 spheroids, which are importantly involved in promoting cancer development and metastasis. Treatment of LECs and LEC + MCF-7 spheroids with estradiol (E2)-induced growth, as well as ERK1/2 and Akt phosphorylation, and was abrogated by miR193a-3p and secretome from MCF-7 transfected cells. Gene expression analysis (GEA) in LEC + MCF-7 spheroids transfected with miR193a-3p showed significant upregulation of 54 genes and downregulation of 73 genes. Pathway enrichment analysis of regulated genes showed significant modulation of several pathways, including interferon, interleukin/cytokine-mediated signaling, innate immune system, ERK1/2 cascade, apoptosis, and estrogen receptor signaling. Transcriptomic analysis showed downregulation in interferon and anti-apoptotic and pro-growth molecules, such as IFI6, IFIT1, OSA1/2, IFITM1, HLA-A/B, PSMB8/9, and PARP9, which are known to regulate BC progression. The cytokine proteome array of miR193a-3p transfected MCF secretome and confirmed the upregulation of several growth inhibitory cytokines, including IFNγ, Il-1a, IL-1ra, IL-32, IL-33, IL-24, IL-27, cystatin, C-reactive protein, Fas ligand, MIG, and sTIM3. Moreover, miR193a-3p alters factors in MCF-7 secretome, which represses ERK1/2 and Akt phosphorylation, induces pro-apoptotic protein and apoptosis in LECs, and downregulates interferon-associated proteins known to promote cancer growth and metastasis. In conclusion, miR193a-3p can potentially modify the tumor microenvironment by altering pro-growth BC secretome and inhibiting LEC growth, and may represent a therapeutic molecule to target breast tumors/cancer.

## 1. Introduction

In women, breast cancer (BC) metastasis represents the most frequent carcinoma and is responsible for the vast majority of deaths [1]. BC progression and metastasis are driven by the active interaction of BC cells with vascular and lymphatic capillaries/endothelial cells, immune cells, as well as tumor microenvironment (TME) cells, i.e., stromal/fibroblasts [2,3,4,5,6]. Apart from the contribution of infiltrating blood vessels in promoting tumor growth, the lymphatic system and factors secreted within the TME actively participate in BC growth [5,7]. Hence a better understanding of events considered hallmarks of cancer growth, including proliferation, apoptosis, and metastasis, is needed in order to define its progression and develop effective treatment [2,3]. Despite therapeutic advances, metastasis still contributes to the majority of deaths from BC [3]. Since both lymphatic and vascular systems participate in BC metastasis [5], a better understanding of their interaction with BC cells and the underlying mechanisms may facilitate the development of effective therapeutic strategies for BC progression [4].

Cross-talk between BC cells and vascular as well as lymphatic endothelium actively participate in the pathophysiology of BC growth/progression, as well as metastasis [5]. Indeed, multiple factors generated within the tumor microenvironment (TME) play an important role in driving BC cancer/tumor growth [3]. Apart from the abnormal growth of cancer cells, vascular angiogenesis/capillary formation in BC tumors, as well as lymphatic activity, facilitates BC progression and metastasis [5,7]. However, individual targeting of the growth of BC cells, BC cells plus vascular angiogenesis, and BC cells plus the lymphatic system resulted in limited success in clinical trials [8,9,10]. This highlights the need for a better understanding of the underlying mechanisms involved. It is feasible that a multipronged approach that targets the growth of cancer cells, pro-growth secretome/TME, as well as angiogenesis and lymphatic growth/activity, may be more effective in inhibiting BC tumor growth [11]. Hence, identifying molecules that can mediate the multipronged inhibitory actions would be of key therapeutic significance. Moreover, developing in vitro models, such as multi-cell spheroids and organoids, would enable a better understanding of the mechanisms driving BC growth and testing the effectiveness of therapeutic molecules. 

Several reports document the involvement of microRNAs (miRNA) in biological processes in breast cancer [12,13]. The miRNAs are small endogenous non-coding RNAs of approximately ~22 nucleotides in length. They bind to the 3′-untranslated regions (UTR) of the target mRNA of protein-coding genes and regulate gene expression. They play an active role in several pathophysiological and biological mechanisms by modulating multiple cellular processes, including cell proliferation, differentiation, apoptosis, invasion, and migration [14]. Increased incidence of miRNA deregulation has been documented in various cancers [15], including BC [13,16], suggesting that miRNAs play essential role(s) in tumorigenesis by regulating the expression of their targets. Numerous miRNAs have been implicated in the pathophysiology of tumor growth (invasion, metastasis, and progression) by modulating suppressor genes and oncogenes [17]. The ability of miRNAs to abrogate multiple growth targets has made them attractive for developing new therapeutic strategies to treat cancer [18,19]. Indeed, multiple miRNA analogs/mimics have shown therapeutic promise and undergoing clinical trials [16,19]. 

In the present study, our focus was to assess the potential role of human miRNA193a-3p in modulating key processes known to drive BC growth, i.e., the growth of lymphatic EC (LECs) and BC secretome. First identified by Lagos-Quintana et al. in 2003 [20], miR193a is a member of the miR193 family located on human chromosome 17q11.2. Other members of the miR193 family include miR193b and miR193c, which can inhibit cell growth by modulating key drivers of the cell cycle [21]. Biogenesis of miRNA leads to the formation of two mature miRNAs (miR193a-3p and miR193a-5p) from pre-miR193a. Importantly, both arms of miRNA-193a are downregulated in various cancers and can trigger growth suppressive actions via different mechanisms. It modulates the expression of specific targets, such as c-kit, uPA, ERBB4, and cyclin D1 [21,22]. It acts as a growth suppressor in both solid [23,24] and liquid tumors [25,26,27]. Synthetic miR193a-3p mimic 1B3 has been shown to induce potent tumor-suppressive and anti-oncogenic actions in multiple cancer cell types [28]. 

We have recently shown that miR193a-3p inhibits the growth of both vascular endothelial cells (VECs) and BC cells (MCF-7; [29]). Moreover, miR193a-3p abrogates BC-driven mitogenic effects on VECs by altering BC secretome [29]. Since LECs actively participate in the pathophysiology of tumor growth/progression and metastasis [5], in the present study, we assessed the effects of miR193a-3p on LEC growth. LECs have been shown to educate BC cells to enhance tumor growth [30] and facilitate metastasis. Hence, targeting LEC growth may further prevent BC progression. Importantly, molecules capable of targeting both VEC and LEC growth may potentially be more potent in abrogating BC growth. LECs originate from a subpopulation of VECs in the cardinal vein or LEC progenitors within the intersegmental vessels during embryogenesis [31]. A key difference between VECs and LECs is that they express PROX-1, which acts as a master control gene for lymphatic differentiation. Additionally, they express VEGFR-3 and LYVE-1 and respond selectively to VEGF-C, VEGF-D, and VEGFR-3. Morphologically, LEC capillaries are diffused with discontinuous junctions without SMCs and pericytes, suggesting that they may be prone to leaks and may play a role in BC metastasis. Although LECs originate from the embryonic subpopulation of VECs, they respond differently to specific growth factors [32]. Similar to VECs and BC cells, whether miR193a-3p inhibits LECs is not known and was investigated.

In ER-positive BC, the growth-promoting role of estrogen is well-established [33]. Estrogen is documented to modulate genes and miRNAs by activating intracellular signaling cascades [34]. Several miRNAs have been shown to play an indispensable role in mediating estrogen responses via various cellular processes in estrogen-responsive cells, such as osteoblasts [35], mesenchymal stem cells [36], osteocytes [37], and VECs [29]. Moreover, miRNAs participate in estrogen regulatory networks [38]. In MCF-7 cells, treatment with E2 increased the expression of 21 miRNAs and decreased the expression of 7 miRNAs [39]. Since miRNAs and E2 play a role in tumor growth, identifying specific E2-regulated miRNAs would provide critical information to improve BC treatment. Interestingly, ectopic expression of miRNAs downregulated by E2 prevents cancer cell proliferation, induces apoptosis, and blocks tumor progression [40]. The importance of miR193a in BC is highlighted by the fact that miRNA193a-3p levels are downregulated in BC [21,41], epigenetic alterations in miR193a-3p induce HER2 positive BC aggressiveness [42], and miR193a abrogates BC proliferation and metastasis [21,43]. We have recently shown that E2-induced growth of ER-positive BC cells (MCF-7) and VECs is accompanied by downregulation of miR193a-3p, and these effects are blocked following ectopic expression of miR193a [29]. Although the impact of E2 on VECs and its relevance in BC is well-established, information on the modulatory actions of E2 and miRNA 193a-3p on LECs is poor. Since the lymphatic system is implicated in BC progression [5,30], miR193a-3p inhibits VEC growth and E2 promotes VEC growth by downregulating its expression [29], together with the fact that LECs evolve from embryonic ECs [31] and promote BC tumor growth. We hypothesize that similar to VECs, E2 promotes LEC growth by lowering miR193a-3p expression. Importantly, ectopic expression of miRNA193a-3p may abrogate the mitogenic actions of E2 in LECs. Since miR193a-3p alters the growth effects of MCF-7 secretome in VECs [29], we hypothesize that miR193a-3p may alter the release of pro- and anti-growth factors in MCF-7 secretome to influence LEC and VEC growth.

As an extension to our previous findings [29], the goal of the present study was to assess the modulatory effects of miRNA193a-3p in regulating the growth of LECs. Using human LECs co-cultured with MCF-7 BC cells in 3D spheroid and LECs in 2D cultures, we assessed the impact of miRNA193a-3p on spheroid and cell growth. We assessed the modulatory effects of miRNA193a-3p on LEC and LEC plus MCF-7 spheroid growth in response to E2 and MCF-7 secretome. Moreover, the impact of miR193a-3p on MCF-7 secretome and E2-induced pro-mitogenic signaling (ERK1/2 and Akt phosphorylation) in LECs and LEC plus MCF-7 spheroids was assessed. Additionally, gene microarrays and transcriptomic analysis were employed to decipher molecular mechanism(s) associated with the growth impact of miR193a-3p on LEC plus the MCF-7 spheroid. Finally, using protein arrays, we assessed changes in growth regulatory molecules secreted by MCF-7 in response to miRNA3a-3p, to better understand and outline mechanisms that may contribute to MCF-7-driven growth of LECs. The above experiments, together with our previous findings [29] are important to define whether miRNA193a-3p mediates its anti-tumor/anti-oncogenic actions via the multipronged inhibition of LEC, VEC, and MCF-7 growth, as well as via alternation of BC secretome. 

## 2. Materials and Methods

### 2.1. Cell Culture

Human lymphatic endothelial cells or dermal microvascular endothelial cells, neonatal and pooled (LECs; >90%), were acquired from Lonza (Walkersville, MD, USA, CC-2516) and grown in the EGM™-2MV BulletKit™ growth medium (Walkersville, MD, CC-3202) containing EBM-2 (endothelial basal medium-2), human epidermal growth factor (hEGF), vascular endothelial growth factor (VEGF), R3-insulin-like growth factor-1 (R3-IGF-1), ascorbic acid, hydrocortisone, human fibroblast growth factor-beta (hFGF-β), fetal bovine serum (FBS), and gentamicin/amphotericin-B (GA). The Reagent Pack (Walkersville, MD, USA CC-5034), containing Trypsin/EDTA, Trypsin Neutralizing Solution, and HEPES Buffered Saline Solution, was used for LEC passaging.

Estrogen responsive MCF-7 human breast cancer cell line (mammary epithelial cells) was provided by Dr. André Fedier (Clinic for Gynecology, University Hospital Zurich). Cells were cultured in a DMEM/F12 medium containing Glutamax (1×), antibiotic-antimycotic (AA 100 μg/mL streptomycin, 100 μg/mL penicillin, and 0.025 μg/mL amphotericin B), and 10% FCS, under standard tissue culture conditions (37 °C, 5% CO_2_).

### 2.2. Conditioned Media (CM) Formation

**Figure 1:**
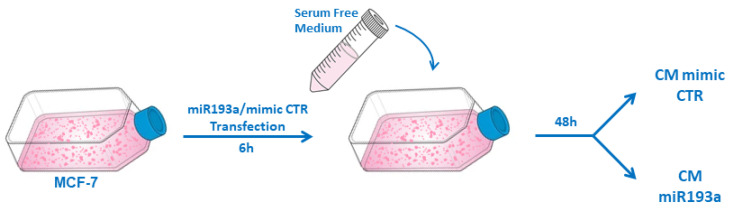
Schematic representation of conditioned serum-free media (CM) formation and collection. MCF-7 cells grown in 75 cm^2^ tissue culture flasks were transfected for 6 h with miR193a or mimic control (CTR). Subsequently, the transfection medium was replaced with the serum-free medium. After 48 h, CM from mimic CTR and miR193a treated cells were collected, centrifuged, and filtered through 0.2 µ syringe filters.

CM from MCF-7 cells was prepared as we previously described [29]. Briefly, MCF-7 cells were cultured in 75 cm^2^ tissue culture flasks and grown to 70% confluency in the growth medium. The cells were washed with HBSS and transfected with miRNA193a-3p or mimic CTR using Lipofectamine 2000. After 6 h the transfection, the medium was aspirated and replaced with a 10 mL serum-free medium (EBM-2, Glutamax, antibiotic-antimycotic solution) for 48 h (Figure 1). Subsequently, the supernatant containing all factors secreted by MCF-7 cells (secretome), was collected, centrifuged (5 min, 1000× *g*, at RT), and filtered through 0.2 µm syringe filters. The resulting conditioned media (CM) was aliquoted and stored at −80 °C until further use.

### 2.3. Spheroid Formation and Culture

Spheroids were prepared as previously described [29,44] using the spheroid microplate method or hanging droplet method. Briefly, for the spheroid formation, 100 µL of the cell suspension containing 5 × 10^2^ cells/well (for proliferation studies) or 5 × 10^3^ cells/well (for Western blot and microarray) were seeded in 96-well U bottom low-attachment plates (Thermo Scientific, Nunclon sphera, 174925). Hanging drops were made by pipetting 15 µL of cell suspension (3.4 × 10^5^ cells/mL) on a Petri dish lid. The lid was then inverted and the cells were forced to accumulate at the bottom of the drop and were allowed to aggregate and form spheroids by incubating for 96 h, under standard tissue culture conditions (humidified atmosphere of 5% CO_2_ at 37 °C). To form multicellular spheroids, we mixed MCF-7 and LECs in a 1:1 ratio.

### 2.4. Transfection with miRNA

Cells cultured in their respective growth medium for 48 h were transfected with mimic negative control and miRNA193a-3p at a final concentration of 25 nM using Lipofectamine2000 (0.17%). Both miRNAs and Lipofectamine2000 were diluted in serum and an antibiotics-free medium (DMEM-F12 (for MCF-7) or EBM-2 (For LECs)). After 5 min incubation at RT, the solutions were mixed in equal volume (a 1:1 ratio) and complex formation was allowed to proceed at RT for 20 min. Cells were washed with HBSS (containing Ca^2+^ and Mg^2+^), rinsed with serum and an antibiotics-free medium, and treated with miRNA:Lipofectamine complex for 6 h. Subsequently, the transfection medium was replaced with a fresh growth/treatment medium. Transfection efficiency of miRNAs in LECs and MCF-7 cells was assessed 48 hours after transfection by detecting fluorescent-labeled miRNA. To obtain transfected spheroids, cells were transfected separately in monoculture, trypsinized, counted, and seeded in a 1:1 ratio. The miR193a-3p used is an hsa-miR193a-3p with the following sequence: 5′–AACUGGCCUACAAAGUCCCAGU–3′.

### 2.5. Cell Proliferation Assay

Cell counting/change in cell numbers was used to assess the proliferation of cell grown in 2D. Cells were plated at a density of 2.5 × 10^4^ cells/well, in 24-well plates, and allowed to attach. Cell growth was arrested by incubating them in a starving medium (EBM-2, Glutamax, antibiotic-antimycotic solution, and 0.4% steroid-free FCS) for 6 h or overnight. Subsequently, cells were treated with CM (supplemented with either 0.4% FCS) or 2.5% steroid-free FCS medium containing or lacking 10 nM E2. After 3 days, the cells were trypsinized and counted using Coulter Counter (Coulter Electronics, Luton, UK). All samples were analyzed in triplicates. The relative cell number was assessed by normalizing it to the control.

The 3D cell proliferation assay was performed by measuring the spheroid area. Spheroids formed in 96-well U-bottom plates with cells pretreated with 10 nM E2 or cells were pre-transfected with miRNA. After 96 h of culture, bright field pictures of spheroids were taken with an inverted microscope. Pictures of spheroids were processed to 300 px/in, using image processing software and analyzed using ImageJ software, as previously described [29,44]. Particles were analyzed and the area was calculated as the number of total pixels.

### 2.6. RT-PCR Gene Expression Studies

To assess gene expression, quantitative PCR was performed using total RNA extracted from cells stimulated with 10 nM E2. RNA extraction was performed using the Quick-RNA MiniPrep Kit (ZymoResearch, CA, USA, R1055), according to the manufacturer’s instructions. Briefly, the cells were dissolved in 300 µL of a lysis buffer, followed by many centrifugation steps, one DNA digestion step, and many washing steps. The purified RNA was eluted with 30 µL DNase/Rnase-free water at top speed. Total RNA was quantified using Tecan Spectrofluorometer (Infinite 200 NanoQuant). The reverse transcription of RNA into cDNA was performed using the TaqMan miRNA Reverse Transcription Kit (Life Technologies, CA, USA (4366597)). The reaction was performed by incubating the mix at 16 °C for 30 min followed by 30 min at 42 °C and inactivation at 85 °C for 5 min. Transcribed cDNA was diluted and mixed with 2× TaqMan Fast Advanced Master mix, 20× TaqMan miRNA Assay mix, DNase/RNase free water, and cDNA. The PCR reaction was run for 2 min at 50 °C and 20 s at 95 °C, followed by 40 cycles of 95 °C for 3 s and 60 °C for 30 s. RNU48 and RNU49 were used as internal controls for miRNA template normalization. The relative gene expression was determined using the 2^−ΔΔCt^ method.

### 2.7. Western Blot

LECs were seeded in 35 mm tissue culture dishes and allowed to adhere and form a monolayer. Subsequently, LECs starved for 7 h were exposed to different CMs (CM miR193a or CM mimic CTR) for 45 min. Next, the cells were rinsed, with ice-cold PBS and lysed on ice. MCF-7 plus LEC spheroids were collected, disaggregated, and lysed in 80 µL of a cell lysis buffer (containing 20 mM Tris pH7.5, 1% Triton X-100, 150 mM NaCl, 1 mM EGTA, 1 mM EDTA, 2.5 mM sodium phosphate, 1 mM β-glycerophosphate, 1 mM sodium vanadate, 0.5 PMSF, and 0.2% SDS). The samples were homogenized for 4 seconds and stored at −20 °C until further use. Protein concentration was measured using the Pierce BCA Assay Kit (Thermo Fisher). The cell extracts/proteins were resolved using 10% SDS-polyacrylamide gel electrophoresis and transferred to a nitrocellulose membrane. The membrane was blocked by incubating in 5% milk and with the primary antibody overnight. After washing 3 times with 1% milk, the membrane was incubated for 1 h with the secondary anti-mouse or anti-rabbit peroxidase-conjugated antibody. The proteins of interest were detected with a CAWOMAT 2000 IR film developer, pre-exposed to a SuperSignal West Dura Luminol Substrate. The band intensity was quantitatively determined using ImageJ software and protein levels were normalized to total protein expression.

### 2.8. Microarray

For microarray analysis, spheroids were harvested, disaggregated, and lysed in 300 µL of a lysis buffer, and total RNA was extracted, as described above (“RT-PCR Gene Expression studies” section above). Affymetrix Clariom S Assay (Applied Biosystems by Thermo Fisher Scientific Inc. OK, USA, 902927) was used for microarray analysis, as described before [29]. Briefly, the hybridization of fragmented biotin-labeled ds cDNA to Clariom™ S arrays (Clariom™ S arrays, human) was used for transcriptome analysis. The Affymetrix Gene-Chip Scanner-3000-7G and GeneChip Command Console Software (GCC) v5.0 were used for scanning, images, and quality control assessments, respectively. In-depth analysis of transcriptome was conducted at the Center for Molecular Medicine Cologne (CMMC) transcriptome facility. Transcriptome Analysis Console (TAC, Applied Bio-systems by Thermo Fisher Scientific Inc. OK, USA) was used to assess differentially regulated genes. For our analysis, we used a fold change cut-off of ±1.5 and an FDR *p*-value of <0.01. The Enrichr website [45] was used to perform pathway analysis. All microarray data are deposited in the public Gene Expression Omnibus (GEO) database (accession No. GSE189084; Available online: https://www.ncbi.nlm.nih.gov/geo/query/acc.cgi?acc=GSE189084) (accessed on 14 July 2022).

### 2.9. Apoptosis Studies

#### 2.9.1. LEC Apoptosis Studies Using the Proteome Profiler Apoptosis Array

Using the Proteome Profiler Human Apoptosis Array Kit (R&D Systems, MN, USA, ARY009), we assessed the modulatory effects of miRNA193a-3p on the expression of 35 apoptosis-related proteins in LECs. Briefly, the LECs were seeded in 60 mm tissue culture dishes (6 × 10^5^ cells/dish) and transfected with either mimic control or miRNA193a-3p (25 nM). After 48 h, the cells were lysed using a lysis buffer, and the protein concentration was quantified using the Pierce BCA Assay Kit (Thermo Fisher). Subsequently, aliquots of LEC lysates containing 185 µg protein were applied to the protein array membranes, and subsequent signals detected on a CAWOMAT 2000 IR film developer (Wiroma AG, Niederscherli, CH, Switzerland), according to the manufacturer’s instructions. The membranes were exposed for 30 s or 1 min and the signal intensity was quantified using ImageJ software. The experiment was performed in duplicate.

#### 2.9.2. LEC Apoptosis Studies using Hoechst 33342 and PI Staining

To assess whether miR193a-3p alters MCF-7 secretome and influences its effects on LECs, cells (LECs) were plated at a density of 5000 cells/well of a black, 96-well clear-bottom microplate. The LECs were cultured for 3 days in CM collected from MCF-7 cells pre-transfected with mimic CTR or miRNA193a-3p in a 37 °C, 5% CO_2_ incubator. The cells were stained with 0.5 µg/ml Hoechst 33342 (blue fluorescent nuclear dye) and 1 mg/mL propidium iodide (PI, red-fluorescent nuclear dye) to determine the proportion of live and dead/apoptotic cells. The cells were incubated for 30 min at 37 °C and analyzed by fluorescence microscopy with a fluorescence inverted microscope (Olympus IX81, Volketswil, CH). Double staining with Hoechst 33342 and PI provides a rapid and convenient assay for apoptosis based upon fluorescent detection for the compacted state of the chromatin in apoptotic cells.

### 2.10. Cytokine Proteome Array in the Secretome

Quantification of cytokines in MCF-7 secretome was performed using the Proteome Profiler Human XL Cytokine Array Kit (R&D Systems, MN, USA, ARY022B). CM from MCF-7 cells was collected, centrifuged, and filtered through a 0.2 µm membrane, and aliquots were frozen at −80 °C. Membranes for cytokine arrays were exposed to 700 µL of CM and incubated overnight, and the proteins were subsequently detected, as per the user’s manual. Amersham Hyperfilm ECL (GE Healthcare Limited, Buckinghamshire, UK) was used in a CAWOMAT 2000 IR film developer (Wiroma AG, Niederscherli, CH, Switzerland) for exposing the membranes. Experiments were performed two times with independently prepared samples. The signal from duplicate spots of two membranes was quantified using ImageJ software and averaged after background subtraction.

### 2.11. Statistical Analysis

R-studio was used for statistical calculations and data are expressed as mean ± SD from three independent experiments conducted in triplicates. A parametric test was performed with ANOVA following proof of normal distribution using the Shapiro–Wilk test; subsequently, Tukey’s HSD multiple pairwise comparisons were conducted. If the normality test failed, we performed a non-parametric test using Kruskal–Wallis rank-sum test and pairwise Wilcoxon test with Benjamini–Hochberg corrections.

## 3. Results

### 3.1. miR193a-3p Inhibits the Growth in LECs and LECs-MCF-7 Spheroids

**Figure 2:**
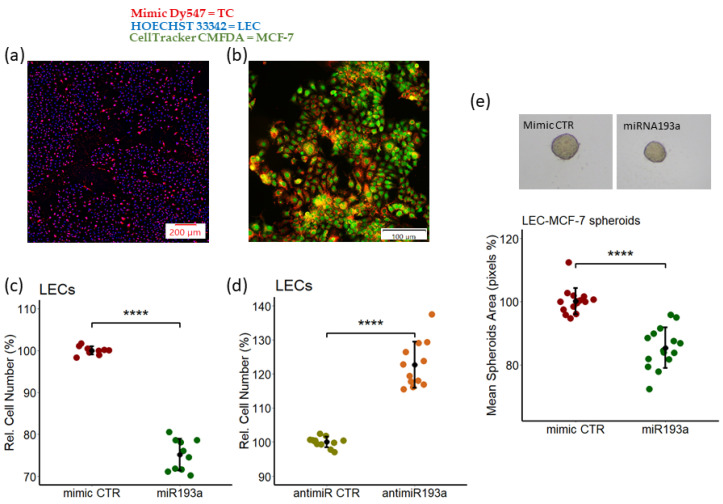
Overexpression of miR193a-3p inhibits LEC proliferation and LEC + MCF-7 spheroid growth. Representative fluorescent photomicrograph showing transfection efficiency of miR193a-3p in LECs (**a**) and MCF-7 cells. (**b**) Red: mimic Dy547; blue: HOECHST 33342; green: CellTracker Green CMFDA; TC: transfected cells. Scale bar: 200 μm (**a**) and 100 μm (**b**). LECs transfected with control (CTR) mimic/miR193a or anti-miRNA CTR/antimiR193a and LEC-MCF7 spheroids were developed using miR193a-3p, and transfected LECs and MCF-7 cells were grown in a 2.5% steroid-free serum-containing medium. After 3 days, growth was assessed by cell counting (**c**) and (**d**) and measuring spheroid area (**e**). Pictures of spheroids were taken on day 4 and analyzed using ImageJ software to calculate spheroid area. Experiments were conducted at least three times in triplicates or quadruplicates. *p* < 0.0001 **** compared to the respective control.

To assess the growth effects of miR193a-3p, we transfected the LECs and MCF-7 cells using our previously established method [29,44] and assessed the transfection efficacy. As shown in Figure 2a,b, a transfection efficiency of >90% was observed in both LECs and MCF-7 cells. To assess the impact of miR193a-3p on growth, LECs transfected with miR193a mimic or anti-miRNA were cultured in a medium containing 2.5% steroid-free serum for 3 days. As shown in Figure 2c,d, transfection with miR193a significantly inhibited LEC proliferation by 25% ± 3.74, whereas miR193a anti-miRNA significantly induced LEC growth by 23% ± 6.8. Since BC-cells interact with LECs to promote BC growth, we assessed the impact of miR193a on the growth of LEC-MCF-7 spheroids. As shown in Figure 2e, compared to spheroids transfected with mimic control (CTR), the serum-induced growth (area) of spheroids transfected with miR193a was inhibited by 16.4% ± 6.4 (*p* < 0.0001). These results demonstrate that miR193a-3p inhibits the growth of LECs, as well as LEC + MCF spheroids.

### 3.2. Modulatory Effects of miRNA193a-3p on ERK-MAPK/PI3K-Akt Phosphorylation in Spheroids

**Figure 3:**
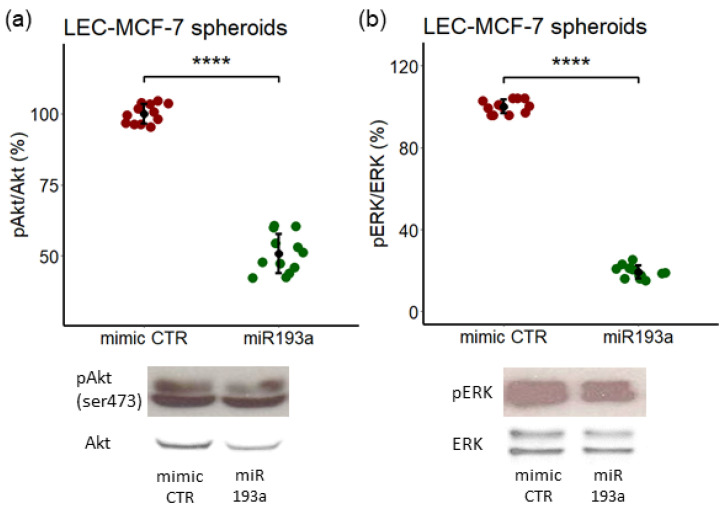
MicroRNA193a (miR193a) inhibits Akt and ERK phosphorylation in LEC + MCF-7 spheroids. Representative Western blots and graphs depicting the inhibition Akt (**a**) and ERK (**b**) phosphorylation in LECs + MCF-7 spheroids transfected with miR193a-3p. Cells were transfected with 25 nM of mimic CTR and miR193a using Lipofectamine2000 and seeded in a U-bottom plate in a 2.5% steroid calf serum medium. Total Akt and ERK were used as the loading control. Western blot analysis was performed on whole spheroid lysates and the band intensity was quantitatively determined using ImageJ software. Experiments were performed at least three times in triplicates and data are represented as mean ± SD. *p* < 0.0001 **** compared to the respective control.

To elucidate the signal transduction mechanisms underlying the growth inhibitory actions in spheroids overexpressing miR193a-3p, we examined the involvement of ERK-MAPK/PI3K-Akt pathways. LEC + MCF-7 spheroids transfected with miR193a-3p or mimic control and cultured for 4 days in a 2.5% steroid-free calf serum medium were lysed, and Akt and ERK phosphorylation were assessed. Both Akt and ERK expression were inhibited by 49% ± 7 and 81% ± 3.1 (Figure 3a), respectively, in spheroids overexpressing miRNA193a-3p. These results suggest that miR193a-3p inhibits LEC + MCF-7 spheroid growth by preventing the activation of ERK-MAPK/PI3K-Akt pathways.

### 3.3. The Effect of Conditioned Media from miR193a-3p Transfected MCF-7 cells on LEC Growth and The Role of ERK-MAPK/PI3K-Akt Pathways

**Figure 4:**
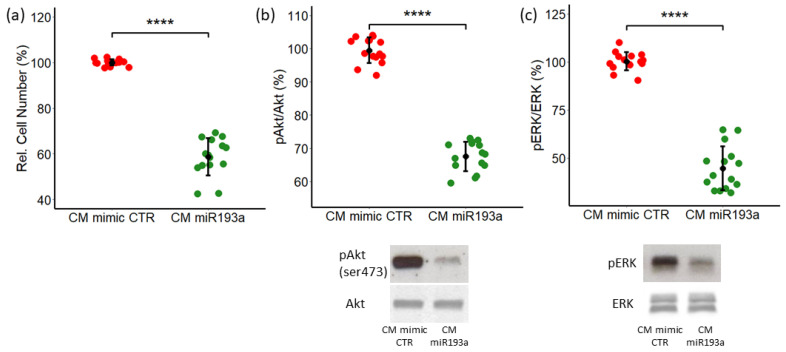
Secretome from miR193a-3p transfected MCF-7 cells inhibits LEC proliferation and AKT, as well as ERK phosphorylation. LECs were cultured in CM miRNA for 48 h and cell proliferation was assessed by cell counting (**a**). Western blotting was performed on the whole cell lysate of LECs exposed to CM miRNA for 45 min. Representative Western blots for AKT (**b**) and ERK ½ (**c**) phosphorylation are depicted in the graph. Total ERK and total AKT were used for normalization. Experiments were performed at least three times in triplicates and data represent mean ±SD. *p* < 0.00001 ****, compared to the respective control.

Recent studies have shown that miRNA193a acts as a tumor suppressor in some malignancies [20,24,46]. To simulate the tumor microenvironment, we collected the secretome/conditioned medium (CM) from MCF-7 cells pre-transfected with either miR193a-3p (CM miR193a) or with the mimic control (CM mimic CTR). We hypothesize that MIR193a-3p CM may alter MCF-7 secretome to inhibit LEC growth. LEC proliferation was investigated in LECs cultured for 48 h in miR193a-3p CM/mimic CTR by cell counting. CM from miR193a-3p transfected MCF-7 cells and significantly inhibited the growth of LECs by 40% ± 8.2 (Figure 4a), suggesting that the soluble factors in CM of miR193a-3p transfected MCF-7 cells and abrogated LEC growth.

To investigate the potential intracellular mechanisms underlying the inhibitory effects of CM from miRNA transfected MCF-7 cells, we evaluated the activation of signal transduction proteins, PI3/Akt and ERK1/2 or MAPK, known to regulate cell proliferation and apoptosis. PI3K/Akt is the most frequently altered pathway in tumors and its activation is associated with cancer progression and tumorigenesis [47,48]. Western blot analysis p-Akt in LECs cultured in miR193a-3p CM showed marked suppression of Akt phosphorylation. Compared to the control CM, CM from miR193a-3p-treated MCF-7 cells inhibited PI3K/Akt phosphorylation by 32.5% ± 4.4 (Figure 4b). Similar to AKT, ERK-MAPK phosphorylation plays an important role in tumorigenesis/cancer and regulates cell proliferation, apoptosis, and migration [49]. Hence, we determined the effects of CM from MCF-7 cells transfected with miR193a-3p on ERK-MAPK phosphorylation in LECs. As shown in Figure 4c, CM from miR193a-3p transfected MCF-7 cells and inhibited ERK phosphorylation by 55% ± 11.4 (Figure 4c).

### 3.4. Estrogen Promotes LEC Growth by Downregulating miRNA193a-3p Expression

**Figure 5:**
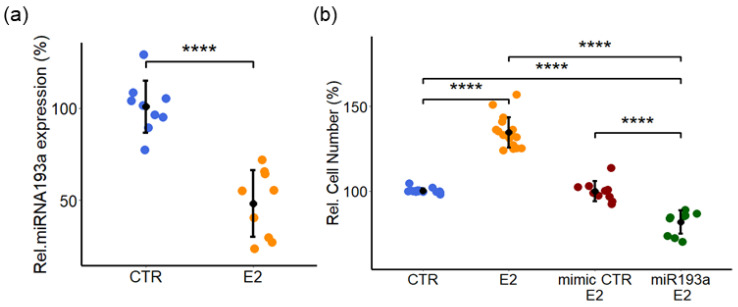
E2 induces LEC growth by downregulating miR193a-3p expression. (**a**) Cells grown in complete media were starved in a steroid-free medium prior to 72 h of treatment with 10 nM E2. Total RNA was extracted and relative miRNA expression levels were determined by RT-qPCR using the TaqMan miRNA assay. The results were normalized to U48 and U49. (**b**) Cells were treated with 10 nM E2 or its vehicle (DMSO) as the control (CTR). Cells were transfected with either the miRNA mimic control or miR193a-3p prior to treatment with 10 nM E2 or the vehicle. Proliferation was assessed by counting LECs. Data represent the mean ± SD. Experiments were performed at least three times in triplicates or quadruplicates. *p* < 0.0005 **** compared to the respective control.

Since E2 and miR193a-3p play important role in cell proliferation, we determined whether the growth effects of E2 on LECs involve miR193a participation. The expression of miR193a-3p in response to E2 was measured in LECs starved in a steroid-free medium and subsequently treated with 10 nM E2 for 72 h. Treatment with E2 inhibited miR193a-3p expression by 40% ± 12.4 in LECs (Figure 5a) and suggested that E2 may regulate LEC growth by downregulating miR193a-3p expression.

To investigate the role of E2 in LECs, we assessed its effects on LEC proliferation and whether ectopic expression of miR193a-3p modulates E2 actions. In LECs treated for 3 days with 10 nM of E2, cell proliferation was induced by 35% ± 9 (Figure 5b). Moreover, ectopic expression of miR193a-3p, but not the mimic control, abrogated the growth stimulatory effects of E2 in LECs, suggesting that E2 may induce LEC growth by downregulating miR193a-3p (Figure 5b).

### 3.5. Effects of E2 on LEC + MCF-7 Spheroid Growth and Activation of ERK-MAPK/PI3K-Akt Pathways: Modulatory Role of miR193a-3p

**Figure 6:**
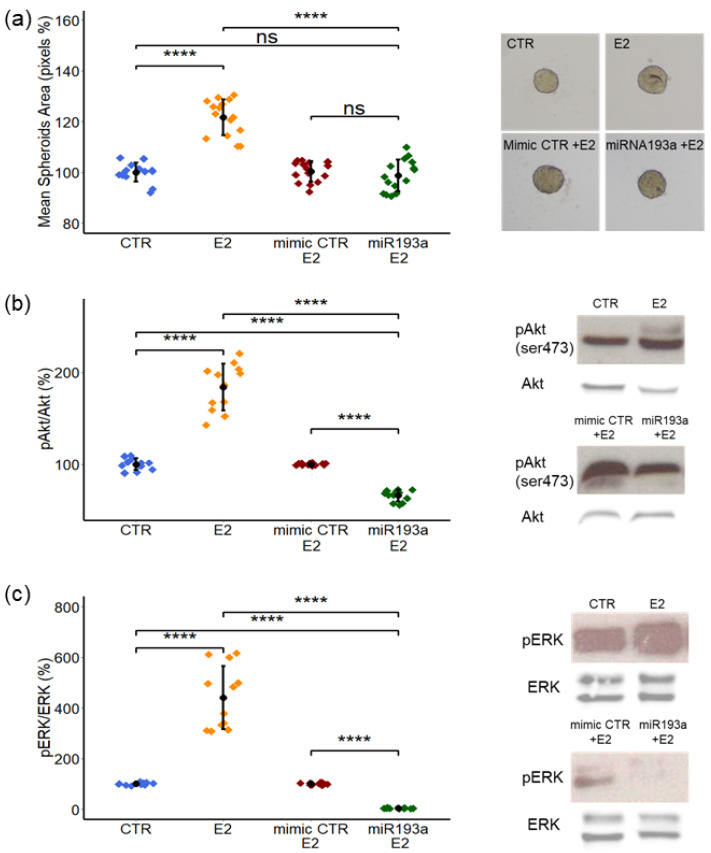
E2-induced spheroid (LEC + MCF-7) growth and signal transduction (AKT, ER1/2 phosphorylation) is abrogated by miR193a. (**a**) Spheroid growth was assessed by pixel analysis of images to calculate the total area of LEC + MCF-7 spheroids treated with the vehicle or 10 nM E2. LEC + MCF-7 spheroids developed using cells transfected with 25 nM of mimic CTR or miRNA193a were exposed to the vehicle or 10 nM E2. Pixel analysis was performed using ImageJ to calculate spheroid area. Representative Western blots and graphs depict AKT (**b**) and ERK (**c**) phosphorylation in lysates from spheroids (mimic control or miR193a) treated with the vehicle (DMSO) or 10 nM of E2/DMSO. The band intensity was quantified using ImageJ software. Phosphorylated protein intensity was normalized to the total protein (ERK and AKT). Data represent the mean ± SD and experiments performed at least three times in triplicates. *p* < 0.00001 **** compared to the respective control.

To assess the effects of E2 on the growth of LEC plusMCF-7 spheroids (a ratio of 1:1), spheroids were grown in 96-well U-bottom plates in the presence of E2 and the respective CTR. After 4 days, the total spheroid area was measured using image analysis. Compared to the vehicle-treated controls, E2 significantly increased growth in LEC + MCF-7 spheroids by 22% ± 7 (Figure 6a). To assess whether miR193a affects E2-induced growth of LEC + MCF-7 spheroids, we tested the effects of E2 in LEC-MCF-7 spheroids developed from cells transfected with the mimic control (CTR) and miR193a-3p. Image analysis of spheroids treated for 4 days with E2 showed that the transfection with miR193a-3p prevented the growth stimulatory effects of E2 (Figure 6a) on LEC + MCF-7 spheroids.

We next determined the role of signal transduction pathways in mediating the growth stimulatory effects of E2 in spheroids by Western blotting. In LEC + MCF-7 spheroids treated with E2 for 4 days, ERK1/2 and Akt phosphorylation was induced by 341% ± 124 and 85% ± 25, respectively (Figure 6b,c). This observation strongly suggests that the growth effects of E2 on spheroids are mediated via the activation of ERK-MAPK/PI3K-Akt pathways.

Since E2 induces LEC growth by downregulating miR193a-3p, we investigated whether it is also involved in modulating the stimulatory actions of E2 on AKT and ERK phosphorylation in spheroids. To achieve our goal, LEC + MCF-7 spheroids transfected with the mimic control or miR193a-3p were cultured for 4 days in an E2 treatment medium. Western blot analysis showed that the stimulatory effects of E2 on AKT and ERK phosphorylation are lost in lysates from spheroids transfected with miR193a-3p (Figure 6b,c). Compared to spheroids transfected with the mimic control, E2 stimulated AKT and ERK phosphorylation was inhibited by 34% ± 6.22 and 97% ± 1.38, respectively, in miR193a-3p transfected spheroids (Figure 6b,c). These results demonstrate that miR193a inhibits LEC + MCF-7 spheroid growth by inhibiting the activation of ERK-MAPK/PI3K-Akt pathways in the presence of E2.

### 3.6. Microarray Analysis

#### 3.6.1. Differentially Regulated Genes

**Figure 7:**
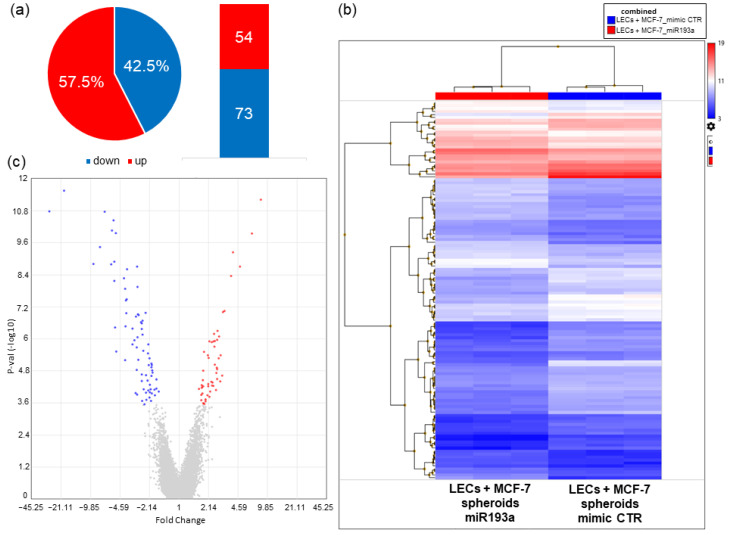
Differentially regulated genes (DRGs) in spheroids transfected with miRNA193a-3p. (**a**) Number of DRGs and a pie chart representation of up and downregulated genes in the percentage of the total number of DRGs. (**b**) Heatmap representation of DRGs in a spheroid formed by LEC + MCF-7 cells transfected with 25 nM of the mimic control and miR193a. (**c**) Volcano plot showing the most upregulated genes (red), the most downregulated genes (blue), and the most statistically significant genes are toward the top. The Transcriptome Analysis Console (TAC, Applied Biosystems) was used for analyzing gene expression data.

We performed microarray analysis to investigate genes associated with the growth inhibitory actions of miR193a-3p on LEC + MCF-7 spheroids. Changes in gene expression were compared in spheroids transfected with miR193a and the mimic control. Change in expression by 1.5-fold and *p* < 0.01 served as cut-off criteria and a total of 127 differentially regulated genes (DRGs) were identified (Supplemental Table S1). Among them, 54 genes were upregulated whereas 73 genes were downregulated using the Transcriptome Analysis Console (TAC) (Figure 7).

A list of the top ten genes up and downregulated in miR193a transfected spheroids are provided in Table 1 and Table 2, respectively.

#### 3.6.2. Pathway Enrichment Analysis of DRGs

**Figure 8:**
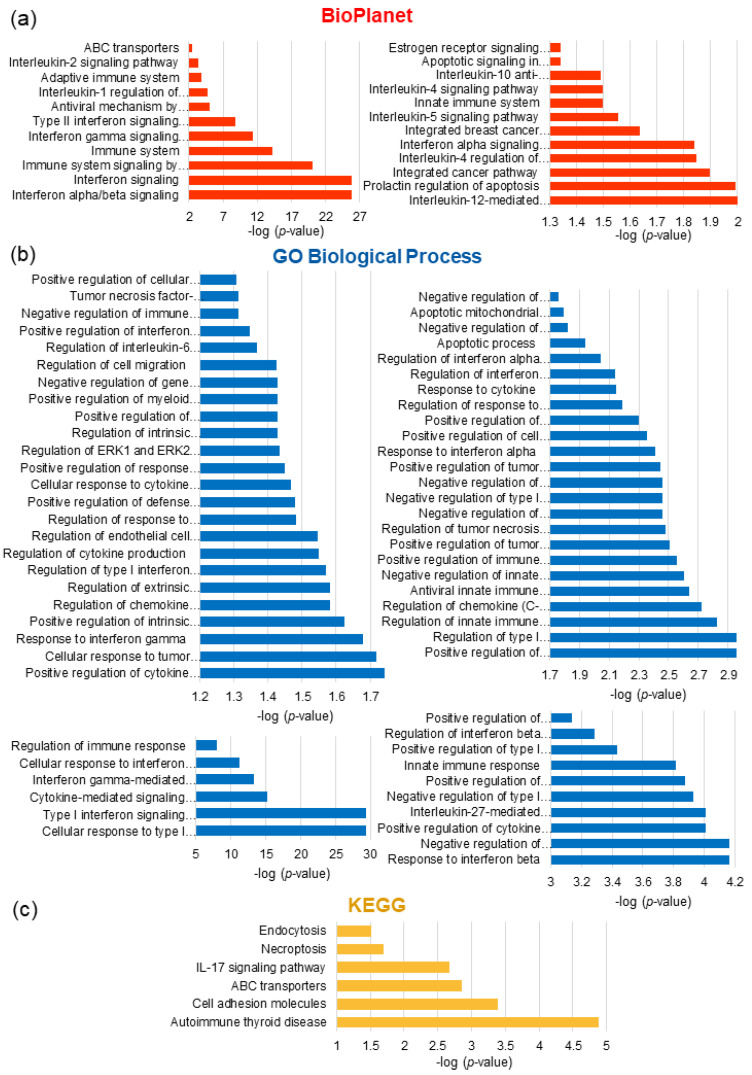
Bar graph showing pathway enrichment analysis of DRGs in spheroids transfected with miR193a and the mimic control. Gene enrichment analysis by (**a**) BioPlanet (red), (**b**) GO Biological Process (blue), and (**c**) the KEGG (yellow; the Kyoto Encyclopedia of Genes and Genomes). *p* < 0.05.

In order to investigate the biological functions of DRGs in spheroids transfected with miR193a-3p and the mimic control, we uploaded the gene list on Enrichr, GO Biological Processes, BioPlanet, and the KEGG. An FDR of <0.05 was used for functional enrichment analysis of differentially expressed genes (Table 3 and Figure 8).

Among the enriched molecular and cellular functions, there were pathways involved in interferon, interleukin/cytokine-mediated signaling, the immune system, as well as regulated ERK1 and ERK2 cascade, apoptosis, ABC transporters, and estrogen receptor signaling pathways. Supplementary Table S2 provides the full list of pathway enrichment analyses.

#### 3.6.3. Gene Identification in Biological Pathways

As mentioned above, DRGs in response to miR193a-3p in LEC + MCF-7 spheroids were especially involved in pathways that play crucial roles in immune response, cell growth, differentiation, as well as motility, and apoptosis. These processes are associated with the expression of various genes, such as members of the interferon family, leukocyte antigen complex, 2′-5′ oligoadenylate synthetases, and proteasome B-type family. Additionally, genes encoding proinflammatory cytokines, signal transducers and activators of transcription, Beta-2-Microglobulin, and enzymes that exhibit phosphohydrolase activity are also expressed (Table 4).

#### 3.6.4. Genes Involved in Apoptotic Pathways

Since pathway enrichment analysis, as well as apoptosis-associated genes, was present in the top ten regulated genes, we further screened the DRGs for apoptosis. Interestingly, the downregulation of anti-apoptotic genes, such as IFI6, was dominant, although several apoptosis-inducing genes, such as S100A8 and AREG, were upregulated. Overall, compared to genes that were upregulated, miR193a-3p overexpression downregulated apoptosis-associated genes by >1.5-fold in spheroids (Table 5). Many of the top ten downregulated genes were also observed in the list of apoptosis-associated genes. 

#### 3.6.5. miRNA193a-3p Induces LEC Apoptosis

**Figure 9:**
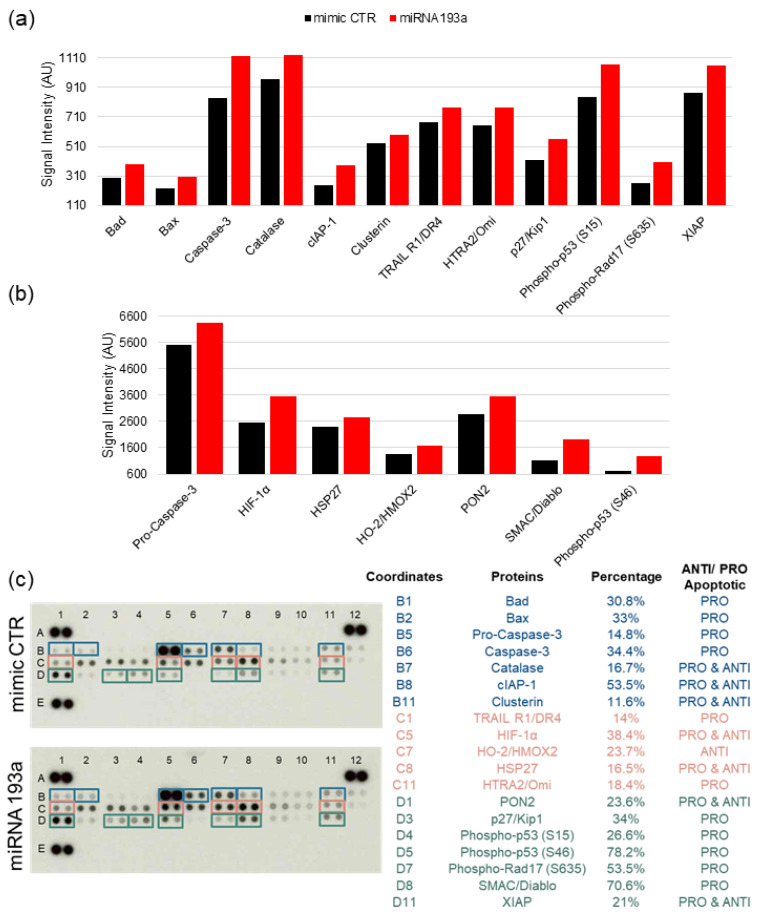
Relative expression of proteins involved in the regulation of apoptosis in LECs transfected with miR193a-3p. Changes in apoptosis-regulating proteins were analyzed using the Proteome Profiler Human Apoptosis Array Kit. Membranes were exposed to lysates (185 µg of total protein) obtained from LECs transfected with mimic CTR or miRNA193a. Bar graphs represent the relative expression of the proteins measured, including expression levels changes between 110 and 1110 AU (**a**), and between 600 and 6600 AU (**b**). Images of nitrocellulose proteome profile membrane showing differences in the expression of apoptosis regulating proteins in lysates of LECs transfected with mimic CTR and miR193a after 1 min of exposure (**c**). The results were analyzed using ImageJ software and depicted final values after background subtraction. The assay was performed using array membranes in duplicate. The legend on the right of the array blots denotes the anti- and pro-apoptotic regulated proteins and the coordinates for their location on the blots in colors.

Both pathway enrichment analysis and DRG analysis of apoptosis-regulated genes revealed that transfection of LEC + MCF-7 spheroids with miR193a-3p significantly modulates apoptosis-associated genes. To substantiate our findings in spheroids, we assessed the impact of miR193a-3p on LEC apoptosis. Compared to LECs transfected with the miRNA control, exposure of apoptosis protein arrays to lysates from miR193a-3p transfected LECs modulated 19 apoptosis-associated proteins by 11.6 to 78.2% (Figure 9). The number of proteins known to mediate pro-apoptotic, dual pro- and anti-apoptotic, and anti-apoptotic actions modulated by miR193a-3p in LECs was eleven, seven, and one, respectively. Overall, miR193a-3p induced pro-apoptotic proteins in LECs by 14% to 78.2% with a collective increase of 425%. Moreover, the ratio between the increase in pro-apoptotic and anti-apoptotic proteins with and without dual action proteins was 17.9-fold and 2.6-fold, respectively. These findings suggest that in LECs, miR193a-3p increases pro-apoptotic proteins and shifts the balance toward apoptosis.

### 3.7. Cytokine Proteome Profiling of MCF-7 Secretome 

**Figure 10:**
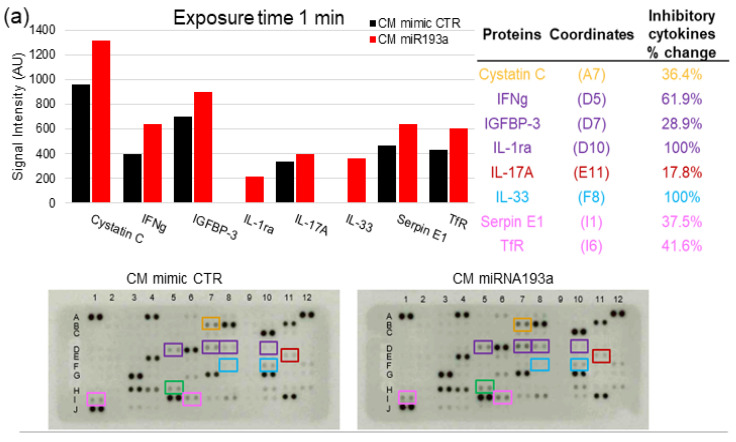

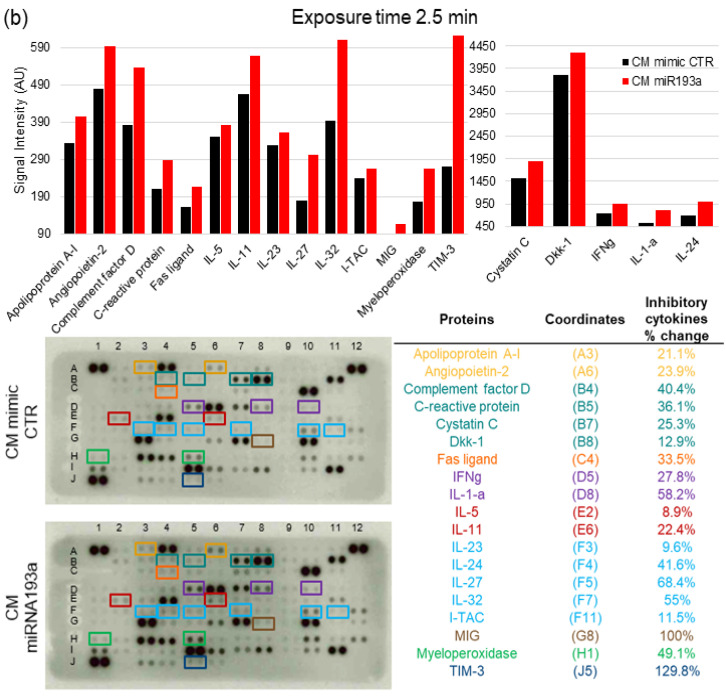
Cytokine(s) expression in MCF-7 secretome is altered by miR193a-3p. Proteome analysis was performed using the Proteome Profiler Human XL Cytokine Array Kit and using equal amounts of conditioned medium (CM) from MCF-7 cells transfected with mimic CTR and miRNA193a (CM miRNA193a). Bar graphs and representative array blots shown are after a short exposure time of 1 min (**a**) and after a long exposure time of 2.5 min (**b**). Images were analyzed using ImageJ after background subtraction. The experiment was performed twice with independent samples. The legend on the right of the array blots depicts inhibitory cytokines and the coordinates for their location on the blots in colors.

Similar to our recent findings in VECs [29], CM from miR193a-3p transfected MCF-7 cells inhibited LEC growth, suggesting that miR193a-3p alters growth-regulating molecules in MCF-7 secretome. To test this, we used a Proteome Profiler Antibody Array Kit for Human Cytokines to determine changes in 105 growth-modulating cytokines in MCF secretome in response to miR193a-3p. To quantify changes in protein expression, we analyzed the means of the pixel number of the pair of duplicate spots and plotted the changes in signal intensity in protein levels (1 or 2.5 min exposure time). We found that miR193a-3p modulated 33 cytokines that negatively influence lymphatic growth (Figure 10). Although an increase in some pro-growth cytokines was observed, the overall increase (%) in antigrowth cytokines was 7.2- and 2.4-fold higher in blots exposed for 1 and 2.5 min, respectively. Figure 10 depicts the growth inhibitory cytokines induced by miR193a-3p in MCF-7 secretome. The growth inhibitory cytokines upregulated by 5–20% were IL-5, IL-23, IL17A, I-TAC, and Dkk-1; by 20–50% were Cystatin C, IGFBP-3, Pentraxin 3, Serpin E1, TfR, Apolipoprotein A-I, Angiopoietin-2, Complement factor D, C-reactive protein, Fas ligand, IL-11, IL-24, and Myeloperoxidase; by 50–90% were IFNγ, IL-1ra, IL-1-a, IL-27, IL-32; and by ≥100% were IL-33, MIG, and secreted/soluble TIM3. These results suggest that miR193a-3p CM inhibits LEC growth by shifting the balance of cytokines toward growth-inhibitory proteins. Importantly, miR193a-3p mainly upregulates anti-angiogenic proteins in MCF-7 secretome.

### 3.8. Secretome from miR193a-3p Transfected MCF-7 Cells Induces Death/Apoptosis in LECs

**Figure 11:**
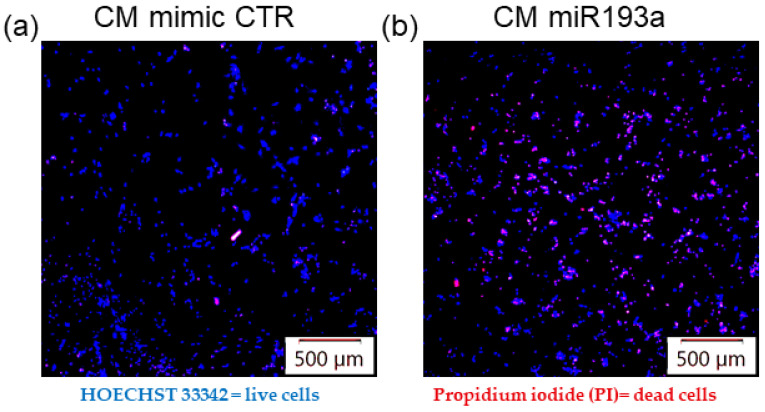
Secretome from miR193a-3p transfected MCF-7 cells induces death/apoptosis in LECs. Representative fluorescent photomicrographs depicting cells stained with propidium iodide (PI; reddish pink) and HOECHST 33342 (blue). Dead or apoptotic cells appear red while living cells appear blue. (**a**) LECs cultured in CM mimic CTR and (**b**) LECs cultured in CM miRNA193a. Live and dead/apoptotic cells were detected by fluorescence microscopy (4×; scale 500 µm); n = 8.

To assess whether miR193a-3p induces changes in MCF-7 secretome and influences LEC survival, we assessed its impact of LEC apoptosis. As shown in the photomicrograph in Figure 11, the number of dead/apoptotic cells (PI positive) was significantly increased in LECs exposed to secretome from miR193a-3p transfected MCF-7 cells, as compared to secretome from cells transfected with the miRNA control. This suggests that miR193a-3p may inhibit the growth of LECs in BC tumors by altering pro-growth or survival factors in MCF-7 secretome.

## 4. Discussion

Lymphatic vessels (LVs) are largely composed of specialized ECs that regulate fluid balance by transporting leaked lymph and cells from blood vessels for recirculation. They not only absorb and transport fats from the intestine and facilitate the host’s immune defense, but also actively participate in the pathophysiology of tumor/cancer growth and progression [30,31,32], as well as in cancer metastasis, including BC [5]. Consistent with the “seed and soil hypothesis” cross-talk between BC cells and LECs, secreted/soluble factor(s) trigger a pro-growth environment in distant organs for BC cells to metastasize [30]. BC cell secretome in the tumor microenvironment (TME) triggers the development of new LVs by promoting LEC growth. Hence, the prevailing view is that drugs that block LV growth may restrict tumor growth/metastasis and be of therapeutic relevance. However, similar to vascular angiogenesis, targeting lymphatic growth has shown partial success against cancer progression [9,10], leading to the notion that multipronged inhibitors, i.e., molecules that target the growth of BC cells, VECs, and LECs, as well as BC secretome, may be more effective in treating BC.

We have recently shown that miR193a-3p, a tumor suppressor miRNA, inhibits MCF-7 BC cell-driven growth of VECs via direct antimitogenic actions and alters MCF-7 secretome [29]. Here, we further investigated the effects of miR193a-3p on LECs and LEC + MCF-7 spheroids and delineated the underlying mechanisms. Transfection of LECs with miR193a-3p, as well as secretome from miR193a-3p transfected MCF-7 cells, inhibited LEC growth, and these effects were mimicked in LEC + MCF-7 spheroids. Moreover, miR193a-3p inhibited ERK1/2 and Akt phosphorylation in LECs and LEC + MCF-7 spheroids, and signal transduction pathways are known to promote cancer development and metastasis. Treatment of LECs and LEC + MCF-7 spheroids with estradiol (E2) induced growth, as well as ERK1/2 and Akt phosphorylation, and these effects were abrogated by miR193a-3p and by secretome from MCF-7 transfected cells. Gene expression analysis (GEA) in LEC + MCF-7 spheroids transfected with miR193a-3p showed 54 genes upregulated and 73 genes downregulated. Pathway enrichment analysis of modulated genes showed significant regulation of several pathways, including interferon (IFN), interleukin/cytokine-mediated signaling, innate immune system, ERK1/2 cascade, apoptosis, and estrogen receptor signaling. Transcriptomic analysis showed downregulation of IFN and anti-apoptotic and pro-growth molecules, such as IFI6, IFIT1, OSA1/2, IFITM1, HLA-A/B, PSMB8/9, and PSMB8, implicated in BC progression. Moreover, the cytokine proteome array of miR193a-3p transfected MCF secretome confirmed the upregulation of several growth inhibitory cytokines, including IFNγ, Il-1a, IL-1ra, IL-32, IL-33, IL-24, IL-27, cystatin, C-reactive protein, Fas ligand, MIG, and sTIM3. Moreover, miR193a-3p modulated the release of factors that represses ERK1/2 and Akt phosphorylation, and downregulated IFN-associated proteins known to induce cancer growth and metastasis. Our findings provide the first evidence that miR193a-3p can potentially modify the tumor microenvironment by altering pro-growth BC secretome and inhibiting LEC growth, and may potentially represent a therapeutic molecule to treat breast tumors/cancer.

LVs actively contribute to BC growth and progression [3,4,5,6]. LVs are largely composed of specialized ECs that differ from vascular ECs, even though they evolve from the same embryonic ECs [31]. LVs are present throughout the body and normally regulate fluid balance by transporting leaked lymph and cells from blood vessels for recirculation, absorbing and transporting fats from the intestine, and facilitating the host’s immune defense [31]. However, under pathological conditions such as cancer, cross-talk between BC cells and LECs via secreted factors trigger pro-growth molecular mechanisms in both BC cells, LECs, and VECs [3,4,5,6]. Hence, molecules capable of blocking this pro-growth cross-talk may be of therapeutic relevance in targeting BC, since miRNA regulates cell function [34] and represents a new class of molecules with therapeutic potential. The fact that miR193a-3p is a tumor suppressor miRNA [46] which is downregulated in BC [21], together with our recent finding that miR193a-3p alters MCF-7 secretome and inhibits mitogen-induced growth of BC cells (MCF-7) and VEC growth [29], we hypothesized that miR193a-3 may also target LEC growth. Our finding that miR193a-3p inhibits MCF-7 secretome-mediated LEC growth suggests that miR193a-3p is a negative regulator of BC growth. The fact that miR193a-3p inhibits the growth of key cells involved in BC progression, i.e., BC cells (MCF-7), VECs, and LECs, implies that it may have the therapeutic capability to inhibit BC progression via a multipronged mechanism. This contention is supported by the recent findings that 1B3, a synthetic miR193 mimic, effectively blocked the growth of multiple cancer cells by targeting key oncogenic pathways [28]. Interestingly, cyclin D, a positive regulator of the cell cycle, is an established target gene for miR193a-3p [26,46].

Our observation that secretome from miR193a-3p transfected MCF-7 cells abrogates LEC growth implies that it alters MCF-7 secretome and may influence the tumor microenvironment (TME), which plays a critical role in driving tumor growth and progression. The breast TME is composed of active molecules generated by multiple cells within its architecture, including cancer cells, fibroblasts/stromal cells, vascular and lymphatic endothelial cells, pericytes, as well as lymphocytes/immune cells [4,5,6]. Importantly, soluble factors and other non-cellular components also play a dynamic role in influencing the growth of both cancer cells, VECs [29,50,51], and LECs [30]. Although communication between different cells within the TME plays an important role in BC, the cross-talk between BC cells and LECs is of particular relevance. The development of LVs by BC secretome in distant organs [5] plays an essential role in BC metastasis by generating the seeding environment for migrating BC cells [5,51]. Moreover, BC-derived factors can educate LECs to promote BC cell growth in a paracrine fashion [5]. Interestingly, MCF-7 secretome also induces proliferation, migration, and capillary formation in VECs [29,52]. Moreover, LEC-derived factors educate BC cells to produce/secrete more growth-promoting molecules in their secretome to enhance tumor growth, as well as increased angiogenesis [51]. The above findings, together with our observation that miR193a-3p alters MCF-7 secretome to abrogate its growth-promoting actions in LECs, suggests that miR193a-3p may, in part, mediate its anti-tumor actions by modulating MCF-7 secretome. Indeed, treatment with miR193a-3p shifted the balance of cytokines in MCF-7 secretome, from pro- to anti-growth by ≥ 2–7-fold.

BC cell secretome contains multiple cytokines that activate LECs [51] and VECs [29] in a paracrine fashion to trigger BC growth and metastasis [5]. Hence, the modulatory effects of miR193a-3p on BC secretome may play an important role in mediating its anti-cancer actions. The contribution of BC-secreted cytokines, such as IL-6, in driving LEC growth via the CCL5/VEGF/Stat3 axis was demonstrated by Lee et al. [5]. Here, we show that the growth inhibitory actions of secretome from miR193a-3p transfected MCF-7 cells are accompanied by increased release of growth inhibitory, anti-angiogenic, and pro-apoptosis cytokines, such as MIG/CXCL9 [53], IL-1a [54], IL-23 [55], IL-27 [56], IL-24 [57], Cystatin C [58], C-reactive protein [59], reduced transferrin receptor (TfR) [60], Serpin E1/PAI-1 [61], and myeloperoxidase [62]. Interestingly we also detected an increase in soluble TIM3, which is postulated to scavenge substrates such as galectin 9, and potentially minimize the pro-growth effects of membrane-bound active TIM3 [63]; however, the importance of this observation needs to be further investigated. The contribution of individual cytokines modulated in secretome in regulating LEC growth can only be speculated and beyond the scope of the present study.

Although abnormal growth of BC cells, vascular angiogenesis, and lymphatic system is a hallmark for BC growth/progression [4,5], molecules targeting cancer/BC cells are not always effective because of cancer cell mutations [1]. Specifically, molecules targeting angiogenesis and lymphangiogenesis also failed in improving BC patient survival [10]. These findings suggest cells of the BC microenvironment, i.e., BC cells, VECs, and LECs may respond differently to factors in TME and drive tumor growth [2]. It is well-established that even though LECs evolve from the same embryonic VECs [3,4,5,6], and selectively respond to different VEGF isoforms. Hence, blockers for VEGF receptors in VECs would be ineffective in LECs and vice versa, and may contribute to the limited clinical success of anti-angiogenesis and lymphangiogenesis therapy. There is increased debate and consensus that a multi-pronged approach of targeting multiple cells or factors that stimulate BC growth may be more effective in treating BC. Our finding that miR193a-3p not only alters MCF-7 secretome from pro- to anti-growth, but also inhibits the growth of MCF-7 [29], VECs [29], and LECs, suggests that it may inhibit BC growth via a multipronged mechanism. Apart from MCF-7, VECs, and LECs, stromal/fibroblasts and other cells are also thought to contribute to BC growth. Hence, further studies to assess the impact of miR193a-3p on key growth-promoting cells in BC tumors is required. A better understanding of the cellular cross-talk mechanism(s) involved would help in developing therapeutic molecules, which can target BC cells and TME in a multi-pronged manner.

E2-induced proliferation and migration of VECs and LECs contribute to vascular and lymphatic infiltration in tumors. BC secretome also stimulates LEC growth in distant organs to serve as a seeding ground for migrating BC cells to promote metastasis [5]. Hence, molecules that interfere with LEC proliferation and migration may prevent BC metastasis. Our finding that miR193a-3p inhibits wound closure and LEC migration suggest that it may be protective against BC metastasis. This notion is supported by the fact that miR193a-3p overexpression inhibits migration of both BC cells [29,43], as well as VECs [29], and alters MCF-7 secretome from pro- to anti-growth in BC cells, VECs, and LECs [29]. Since miR193a-3p targets cyclin D1, a positive regulator of cell cycle, has tumor suppressive actions in various cancers [46], and its expression is reduced in BC [21,29], suggests that it may be effective in inhibiting BC growth. Interestingly, miR193a-3p mimic 1B3 suppresses tumor function/growth [28]. Although promising, additional in-depth studies are required to confirm the anti-metastatic role of miR193a-3p.

Multicell 3D spheroids represent a more realistic model to study cell–cell interaction in cancer/tumor [44] and reflect TME. Using VEC + MCF-7 spheroids, we have previously established a viable model to assess the inhibitory effects of miR193a-3p on their growth and the underlying molecular mechanisms [29]. Here, we used LEC + MCF-7 spheroids to assess the impact of miR193a-3p on their interactive growth and the underlying molecular mechanisms. Our finding that FCS-induced growth of LEC + MCF-7 spheroids was abrogated by ectopic expression of miR193a-3p suggests that it may prevent growth processes driven by MCF-7-LEC interaction in BC growth. In this context, Lee et al. have shown that secretome from BC cells and TME can trigger lymphangiogenesis in distant organs and create an optimal environment for migrating BC cells to seed and grow [30]. Moreover, using VEC + MCF-7 spheroids, we have previously shown it to be a viable model to study the growth effects of specific cell–cell interaction in BC.

The role of estrogen in the pathophysiology of estrogen-sensitive breast cancer is well-established [33]. Since MCF-7 cells represent ER-positive BC cells, we assessed whether miR193a-3p negates E2-induced growth in LECs and LEC + MCF-7 spheroids. Our observation that similar to MCF-7 cells and VECs [29]. The growth stimulatory effects of E2 in LECs were accompanied by the downregulation of miR193a-3p expression, suggesting that it may represent a key mechanism mediating the mitogenic actions of E2 in BC. This contention is supported by the observation that similar to MCF-7 and VECs, the pro-growth effects of E2 in LECs and LEC + MCF-7 spheroids were abrogated following ectopic expression of miR193a. Interestingly, miR193a-3p levels decreased in BC [21], and estrogen regulates miR193a-3p levels in humans [34]. Although miR193a-3p inhibits E2-induced growth, its antigrowth effects are not limited to E2, as it also inhibits growth induced by the serum that contains a battery of growth factors. Altogether, our observations suggest that miR193a-3p negatively regulates MCF-7, VEC, and LEC growth and may antagonize the growth stimulatory effects of E2 in BC via a multipronged fashion by targeting BC cells, vascular angiogenesis, as well as the lymphatic system.

The activation of signal transduction pathways PI3K/AKT [47] and MAPK/ERK1/2 [48] is linked to BC progression and metastasis. Molecules that target and inhibit Akt and ERK activity prevent tumor growth and metastasis [64,65]. Our finding that serum and E2-induced ERK1/2 and Akt phosphorylation was inhibited in LEC + MCF-7 spheroids overexpressing miR193a-3p, as well as in LECs treated with condition medium from miR193a-3p transfected MCF-7 cells, suggests that miR193a-3p not only inhibits ERK1/2 and Akt directly but also abrogates the release of ERK and Akt activating proangiogenic factors secreted by BC cells. Consistent with the above findings, miR193a-3p also inhibits ERK1/2 and Akt phosphorylation in MCF-7 cells and VECs [29]. Since ERK1/2 and Akt inhibitors are of clinical relevance for treating BC progression [64,65], miR193a-3p may represent a viable therapeutic molecule due to its dual inhibitory actions.

To identify the molecular mechanisms influenced by miR193a-3p, we used a microarray approach and investigated the transcriptome profile in LEC + MCF-7 spheroids with the ectopic expression of miR193a-3p or the control vector. In DRG analysis, we identified 54 upregulated and 73 downregulated genes in spheroids with the ectopic expression of miR193a-3p. Functional annotation results of the data revealed that DRGs in defense/innate immune response, viral processes, cell proliferation, viability, differentiation, and apoptosis were mainly involved. To gain insight(s) into the interaction of the DRGs, we performed the GO Biological Process, BioPlanet, and the KEGG pathway analysis. They highlighted that DRGs that were significantly enriched represented type I and II IFN and cytokine signaling pathways. Since many DRGs participate in immune response, tumor growth inhibition, and apoptosis [66,67], it is feasible that miR193a-3p inhibits LEC + MCF-7 spheroid growth via IFN signaling mechanism(s).

Among the most significantly enriched pathways involved were IFN alpha/beta signaling (BioPlanet, adjusted *p*-value 1.22 × 10^−26^) and cellular response to type I IFN (GO 0071357, adjusted *p*-value 4.66 × 10^−30^). Type I IFN, including IFN-α and IFN-β, mediate contradictory effects. On one side they directly promote antitumor response by suppressing tumor growth and on the other side they induce tumor progression by establishing pro-growth conditions in the tumor microenvironment [67,68]. The DRGs of IFN α/β signaling and cellular response to type I IFN, which were downregulated, include genes involved in tumor progression (SAMHD1 [69], PLSCR1 [70], IFITs (IFIT1, IFIT2, and IFIT3 [71]), TAPs (TAP1 and TAP2) [72], cell proliferation, apoptosis/cell survival, metastasis, angiogenesis (PSMB8 [73], PSMB9 [74], IFI6 [75], and IFI27 [76]), OAS’s (OAS1, OAS2, and OAS3) [77], IFITMs (IFITM1, IFITM2, and IFITM3) [78,79], UBE2L6 [80], USP18 [81], tumorigenesis and genes with a role in cancer STAT1 [82], RSAD2 [83], IFI6 [84], and HLAs-class-I (HLA-A, HLA-B, HLA-C, HLA-F, and HLA-G) [85]). The upregulated genes (by ≥ 1.5-fold) involved in apoptosis and autophagy, cell death, and cell growth inhibition were S100A8 and S100A9 [86], MMP1 [87], MMP13 [88], AREG [89], PLIN2 [90], and AKR1C1 [91]. Although, some anti-apoptotic genes were also upregulated (HMOX1, S100A7, CEMIP, and CA9). Since miR193a-3p inhibits the growth of LECs, VECs, MCF-7 cells, and spheroids, the observed upregulation may be due to hypoxic conditions in spheroids. The present analysis highlights that the expression of many growth inhibitory genes, which target apoptosis, cell proliferation, and migration processes, were highly modulated in LEC + MCF-7 spheroids transfected with miR193a-3p.

In LEC + MCF7 spheroids transfected with miR193a-3p, the magnitude of change for the top 10 downregulated genes (2.41–4.84-fold) was much higher than that of upregulated genes (1.53–3.06-fold), suggesting that impact of the downregulated gene is of greater significance in modulating spheroid function. Many of the genes upregulated, i.e., MMP1, MMP13, S100A7, CEMIP, and CA9 are known to increase in various cancer cells. Interestingly, the genes upregulated in LEC + MCF-7 spheroids in response to miR193a-3p were not the same as those that were upregulated in VECs, suggesting that miR193a-3p influences LECs and VECs differentially. Since ectopic expression of miR193a-3p in LECs, as well as LEC + MCF-7 spheroids, inhibited proliferation/migration and spheroid growth, respectively, the pro-growth genes that were upregulated (marginally) may be a response to hypoxic conditions in spheroids or have roles other than growth regulation. The most prominent group from the downregulated genes were IFN-associated genes, i.e., IFI6, IFIT1, OAS1, OAS2, PSMB9, HLA-B, HLA-A, IFITM1, PARP9, and PSMB8. Interestingly all these genes, except PSMB8, are also downregulated by miR193a-3p in VECs [29]. Our findings suggest that miR193a-3p inhibits LEC + MCF-7 spheroid growth by downregulating pro-growth and cancer-inducing genes.

In cancer pathophysiology, a long-standing view is that IFN inhibits both angiogenesis and cancer cell growth. However, more recent studies provide evidence that IFN can also mediate pro-tumorigenic effects [71]. Functional studies with IFIT proteins (IFIT1 and IFIT3) on various molecular signaling mechanisms implicates them in cancer progression and metastasis [92]. Interestingly, the pro-cancer growth effects of IFIT proteins involve Akt activation and were downregulated in miR193a-3p transfected LEC + MCF-7 spheroids. We observed similar effects on VECs exposed to secretome from MCF-7 cells transfected with miR193a-3p [29], suggesting that miR193a-3p inhibits spheroid growth by preventing MCF-7–LEC cross-talk by potentially downregulating IFIT proteins and inhibiting Akt [92]. Interestingly, in SUM149 inflammatory BC, the overexpression of IFITM1 enhances aggressive phenotype via signal transducer and activator of transcription 2 (STAT2) [93]. Moreover, IFN-stimulated genes are downregulated by progesterone, which counteracts the growth-promoting actions of estrogen in BC [94]. Our findings that miR193a-3p inhibits spheroid growth and downregulates IFN-associated proteins and signaling suggest that it inhibits growth by specifically downregulating pro-growth actions of IFN.

Our finding that IFN-associated genes were strongly downregulated in miR193a-3p transfected LEC + MCF-7 spheroids was also observed previously in VECs [29] and was unexpected. Since miR193a-3p inhibits spheroid growth and also downregulates genes for multiple IFN-induced transmembrane protein-inducing IFITM1, IFIT1, IFIT3, as well as IFN pathways, it implies they may play a prominent role in inhibiting the growth of LEC + MCF-7 spheroids by modulating MCF-7 and LEC cross-talk. However, in-depth studies are required to confirm this notion. In ECs, there is a high expression of IFITM1 during sprouting and lumen formation, and these processes are disrupted by IFITM1 knockdown [79,95]. It is postulated that IFITMs regulate the transition of quiescent ECs toward an angiogenic state [79,95]. Although the role of IFITM1 in VECs is well-established, its role in LECs is unclear. Since LECs are derived from VECs [3,4], it is feasible that IFITM1 has a similar role in LECs. Interestingly, IFIT1 and IFIT3 genes are upregulated during cancer metastasis and invasion [71] and mediate their growth-promoting actions by complexing and phosphorylating Hsp90 and its client proteins, including PKC, EGFR, Akt, and p38 [71]. These characteristics, along with our observation that miR193a-3p inhibits LEC + MCF-7 spheroid growth, as well as Akt and ERK1/2 phosphorylation, implies that it may inhibit MCF-7 and LECs growth, in part, via this mechanism. Interestingly, ectopic expression of IFIT1 and IFIT3 enhances many EGFR and annexin-2 pathways to induce cell proliferation, survival, and drug resistance [71]. Moreover, USP18, which mediates angiogenic effects (sprouting and capillary formation) of IFN-α in ECs [96,97], was downregulated by miR193a-3p in spheroids. The above findings, together with the fact that USP18 deficiency creates antitumor activity in mammary cells [98], suggest that miR193a-3p may prevent MCF-7 and LEC cross-talk and abrogate IFN-driven angiogenic processes in BC, inhibiting growth.

The top ten genes that were downregulated in miR193a-3p transfected LEC + MCF-7 spheroids were also OAS1 and OAS2. They are members of the IFN-induced enzyme family which convert ATP to 2′,5′-linked oligomers of adenosine, which inhibits the proliferation of the RNA virus. Increased expression of OAS genes is observed in various cancers, including BC [77]; however, not much is known about its role in the lymphatic and vascular systems. Our finding that OAS1 and OAS2 were downregulated in LEC + MCF-7 spheroids transfected with miR193a-3p and similar effects were previously observed in VECs exposed to secretome from miR193a-3p transfected MCF-7 cells [29], suggesting that miR193a-3p modulates the release of MCF-7-derived factors that inhibit the innate immune response by ECs, resulting in OAS1 and OAS2 downregulation. Since increased expression of OAS1 and OAS2 is linked to BC, downregulation by miR193a-3p implies that it may mediate its antitumoral activity, in part, by downregulating OAS1 and OAS2. However, in-depth studies are required to test this hypothesis.

Anti-apoptotic effects of IFNs play an important role in BC growth and progression [99]. IFI6 or GIP3 is an antiapoptotic protein localized in mitochondria and promotes BC progression by generating mitochondrial (mt) ROS [75], inhibiting IFI6-induced mtROS is of therapeutic relevance in preventing BC migration and metastasis. In LEC + MCF-7 spheroids, overexpression miR193a-3p IFI6 was the top downregulated gene (−4.84 fold), suggesting that miR193a-3p may inhibit BC growth by abrogating the antiapoptotic actions of IFI6. Interestingly, miR193a-3p also downregulated the expression of IFI27, an IFNα-inducible oncogenic protein highly expressed in cancer that promotes cell migration by reducing VDGF-A and actin filaments [100]. Since IFI6 and IFI27 are downstream targets of activating transcription factor-3 and suppressed to mediate its growth inhibitory actions in cancer cells [101], it is feasible that the same mechanism mediates the growth inhibitory actions of miR193a-3p in spheroids and needs to be investigated.

Apart from the IFN-associated mechanisms that regulate growth, the overexpression of miR193a-3p downregulated PARP9, a member of poly(ADP-ribose) polymerase family member 9, which is overexpressed in human BC cells and promotes cell migration [102]. Moreover, PARP9 expression positively correlates with metastasis of axillary lymph node and is negatively associated with ER expression [102], as well as PSMB8 and PSMB9 and immunoproteasomes, which induce tumor angiogenesis via VEGF-A and are overexpressed in most cancers, including BC [73,74]. Moreover, miR193a-3p downregulated the expression of three major histocompatibility complex class proteins (HLA-A, HLA-B, and HLA-C) which are known to interact with integrin-β4 on the endothelial cell surface and promote growth [85]. Interestingly, the pro-growth signaling involves Akt and ERK activation/phosphorylation [85], which are inhibited in LECs and spheroids by miR193a-3p. Since ECs in general and LECs are postulated to respond as modified immune cells, together with the above findings, it is feasible that miR193a-3p inhibits the innate immune response of LECs to MCF-7-derived factors and inhibits growth. Additionally, miR193a-CM upregulated genes for S100A8, AKR1C1, AREG, and PLIN2 [86,89,90,91] were shown to inhibit angiogenesis and cancer growth. Interestingly, the genes downregulated in VEC by CM from miR193a-3p transfected MCF-7 cells [29] were also downregulated in LEC + MCF-7 spheroids overexpressing miR193a-3p, suggesting that they play a key role in mediating the growth inhibitory actions of miR193a-3p. Moreover, the genes that were upregulated by miR193a-3p in VECs and spheroids were different. Taken together, the above findings suggest that as in VECs, miR193a-3p inhibits LEC + MCF-7 growth by switching the release of soluble factors from MCF-7 cells from pro- to anti-LEC growth.

Since miR193a-3p strongly downregulated anti-apoptosis genes, such as IFI6 in spheroids, and this was also reflected in pathway enrichment analysis, we screened for apoptosis-associated genes that were modulated in spheroids by miR193a-3p. The fact that miR193a-3p downregulated several anti-apoptosis genes and upregulated pro-apoptotic genes suggests that it may mediate its antitumoral activity in BC by triggering apoptosis in cells that contribute to the BC tumor microenvironment. Consistent with this notion, results from the apoptosis proteome array of miR193a-3p transfected LECs showed increased expression of pro-apoptotic molecules (Bad, Bax, Pro-Caspase3, Caspase3, TRAIL R1, p27/Kip1, phospho-p53 (S15 and S46), phospho Rad17 (S635), and SMAC/Diablo). Correctively, the pro-apoptotic proteins increased by ~425% and by ~2.5-fold compared to anti-apoptotic proteins. Moreover, the proteome array of cytokines in the secretome of miR193a-3p transfected MCF-7 showed upregulation of several growth inhibitory and apoptosis-inducing cytokines, such as Fas ligand [103], IL27 [56], IL23 [55], IL24, IL-1a [54], IFNγ [104], CXCL9/MIG [53], C reactive protein [59], cystatin C [58], complement factor D [105], myeloperoxidase [62], IL32 [106], IL33 [107,108], IL-1αRA [109], and I-TAC [110]. Interestingly we also observed increased cell death/apoptosis in LECs exposed to CM from miR193a-3p transfected MCF-7cells; moreover, miR193a-3p downregulates the same anti-apoptotic genes in LEC + MCF-7 spheroids and VECs [29]. However, in-depth studies are required to confirm this contention.

There are several limitations of the present study that should be addressed in the future. Our observation that in LECs, miR193a-3p inhibits growth effects of MCF-7 secretome, which in-itself attenuates LEC growth [52], suggests that miR193a3p enhances the growth attenuating actions of MCF-7 secretome. Hence, the inhibitory actions of miR193a-3p on LEC growth should be confirmed using secretomes from BC cells, which stimulate LEC growth, i.e. MDA-MB231 [30]. Although we provide evidence that miR193a-3p mediates growth inhibitory actions in LEC + MCF-7 spheroids and modulates several genes, the contribution of the specific cell type remains unknown. Moreover, which cross-talk factor(s) mediate the overall actions in spheroids have not been identified. The pro-apoptotic role of DRGs that are significantly regulated by miR193a-3p in spheroids should be further elucidated using molecular gain or loss of function approaches. Whether the selected DRGs are modulated by ERK/Akt and E2 and its receptors, particularly ER-α, may provide insights into their regulatory role in BC secretome-driven LEC growth. Finally, how miR193a-3p modulates cross-talk between MCF-7 and LECs in spheroids should be investigated by selectively targeting MCF-7 or LECs in LEC + MCF-7 spheroids. 

## 5. Conclusions

In conclusion, our findings provide the first evidence that miR193a-3p inhibits MCF-7 secretome and E2-induced growth of LECs, as well as LEC + MCF-7 spheroids. Importantly miR193a-3p alters the profile of growth modulatory cytokines/factors in MCF-7 secretome. At a molecular level, miR193a-3p inhibits Akt and ERK phosphorylation, downregulates pro-angiogenic, as well as anti-apoptotic IFN-associated genes, and increases pro-apoptotic proteins. These results, together with our previous finding in VECs [29], suggest that miR193a-3p may abrogate BC growth by targeting a key component of BC TME, i.e., the growth of BC cells, LECs, VECs, and BC secretome. In conclusion, miR193a-3p may represent a promising therapeutic molecule against BC.

## Figures and Tables

**Table 1:** Top ten upregulated genes in spheroids transfected with miR193a.

Gene Symbol	Description	Log2 FC	FDR *p*-Value
MMP1	matrix metallopeptidase 1	3.06	6.61 × 10^−8^
S100A7	S100 calcium-binding protein A7	2.73	3.08 × 10^−7^
S100A8	S100 calcium-binding protein A8	2.26	2.83 × 10^−6^
CEMIP	cell migration-inducing protein, hyaluronan binding	2.01	1.25 × 10^−6^
MMP13	matrix metallopeptidase 13	1.93	5.61 × 10^−6^
AKR1C1	aldo-keto reductase family 1, member C1	1.7	8.07 × 10^−5^
CA9	carbonic anhydrase IX	1.63	8.52 × 10^−5^
AREG	amphiregulin	1.61	0.0065
HMOX1	heme oxygenase 1	1.55	0.0016
PLIN2	perilipin 2	1.53	0.01

The Transcriptome Analysis Console (TAC, Applied Biosystems) was used for analyzing gene expression data of spheroids transfected with miR193a and the respective mimic CTR. The table lists the top ten upregulated genes with the respective fold changes (FC) and adjusted *p*-values (FDR value). For the analysis, a fold change (FC) cut-off of +/−1.5 and an FDR *p*-value of 0.01 were applied.

**Table 2:** Top ten downregulated genes in spheroids transfected with miR193a.

Gene Symbol	Description	Log2 FC	FDR *p*-Value
IFI6	interferon, alpha-inducible protein 6	−4.84	9.24 × 10^−8^
IFIT1	interferon-induced protein with tetratricopeptide repeats 1	−4.29	5.98 × 10^−8^
OAS1	2-5-oligoadenylate synthetase 1	−3.20	2.65 × 10^−6^
PSMB9	proteasome subunit beta 9	−2.95	8.75 × 10^−7^
HLA-B	major histocompatibility complex, class I, B	−2.78	9.24 × 10^−8^
OAS2	2-5-oligoadenylate synthetase 2	−2.53	2.65 × 10^−6^
HLA-A	major histocompatibility complex, class I, A	−2.51	3.08 × 10^−7^
IFITM1	interferon induced transmembrane protein 1	−2.44	1.58 × 10^−7^
PARP9	poly(ADP-ribose) polymerase family member 9	−2.42	7.67 × 10^−6^
PSMB8	proteasome subunit beta 8	−2.41	2.52 × 10^−6^

The Transcriptome Analysis Console (TAC, Applied Biosystems) was used for analyzing gene expression data of spheroids transfected with miR193a. The table lists the top ten downregulated genes with the respective fold changes (FC) and adjusted values (FDR *p*-value). For the analysis, a fold change (FC) cut-off of +/−1.5 and an FDR *p*-value of 0.01 were applied.

**Table 3:** Pathway enrichment analysis of DRGs in spheroids transfected with miR193a and the mimic control.

Pathway	Overlap	Adj. *p*-Value
**BioPlanet**		
Interferon alpha/beta signaling	20/64	1.22 × 10^−26^
Interferon signaling	26/168	1.22 × 10^−26^
Immune system signaling by interferons, interleukins, prolactin, and growth hormones	26/280	6.36 × 10^−21^
Immune system	35/998	4.68 × 10^−15^
Interferon gamma signaling pathway	13/97	3.76 × 10^−12^
Type II interferon signaling (interferon gamma)	9/50	1.37 × 10^−9^
Antiviral mechanism by interferon-stimulated genes	7/70	8.22 × 10^−6^
Interleukin-1 regulation of extracellular matrix	8/120	2.13 × 10^−5^
Adaptive immune system	15/606	1.29 × 10^−4^
Interleukin-2 signaling pathway	17/847	4.18 × 10^−4^
ABC transporters	4/47	0.0032
Interleukin-12-mediated signaling events	04/65	0.0094
Prolactin regulation of apoptosis	5/118	0.0102
Integrated cancer pathway	3/35	0.0127
Interleukin-4 regulation of apoptosis	7/267	0.0142
Interferon alpha signaling pathway	2/10	0.0145
Integrated breast cancer pathway	5/152	0.0230
Interleukin-5 signaling pathway	3/49	0.0278
Innate immune system	7/319	0.0316
Interleukin-4 signaling pathway	4/104	0.0316
Interleukin-10 anti-inflammatory signaling pathway	2/16	0.0322
Apoptotic signaling in response to DNA damage	2/20	0.0456
Estrogen receptor signaling pathway	2/20	0.0457
**GO Biological Process**		
Cellular response to type I interferon (GO:0071357)	22/65	4.66 × 10^−30^
Type I interferon signaling pathway (GO:0060337)	22/65	4.66 × 10^−30^
Cytokine-mediated signaling pathway (GO:0019221)	30/621	4.83 × 10^−16^
Interferon gamma-mediated signaling pathway (GO:0060333)	13/68	5.37 × 10^−14^
Cellular response to interferon gamma (GO:0071346)	14/121	5.05 × 10^−12^
Regulation of immune response (GO:0050776)	13/179	1.06 × 10^−8^
Response to interferon beta (GO:0035456)	5/28	4.74 × 10^−5^
Negative regulation of cytokine-mediated signaling pathway (GO:0001960)	6/55	6.89 × 10^−5^
Positive regulation of cytokine production involved in immune response (GO:0002720)	5/33	9.83 × 10^−5^
Interleukin-27-mediated signaling pathway (GO:0070106)	4/15	9.83 × 10^−5^
Negative regulation of type I interferon-mediated signaling pathway (GO:0060339)	4/16	1.18 × 10^−4^
Positive regulation of interferon beta production (GO:0032728)	5/36	1.32 × 10^−4^
Innate immune response (GO:0045087)	11/302	1.53 × 10^-4^
Positive regulation of type I interferon production (GO:0032481)	6/77	3.67 × 10^−4^
Regulation of interferon beta production (GO:0032648)	5/49	5.17 × 10^−4^
Positive regulation of chemokine production (GO:0032722)	5/53	7.26 × 10^−4^
Positive regulation of apoptotic signaling pathway (GO:2001235)	5/59	0.0011
Regulation of type I interferon-mediated signaling pathway (GO:0060338)	4/30	0.0011
Regulation of innate immune response (GO:0045088)	5/64	0.0015
Regulation of chemokine (C-X-C motif) ligand 2 production (GO:2000341)	3/13	0.0019
Antiviral innate immune response (GO:0140374)	3/14	0.0023
Negative regulation of innate immune response (GO:0045824)	4/38	0.0025
Positive regulation of immune response (GO:0050778)	5/75	0.0028
Positive regulation of tumor necrosis factor production (GO:0032760)	5/77	0.0031
Regulation of tumor necrosis factor production (GO:0032680)	6/124	0.0033
Negative regulation of extrinsic apoptotic signaling pathway (GO:2001237)	5/80	0.0035
Negative regulation of type I interferon production (GO:0032480)	4/43	0.0035
Negative regulation of chemokine production (GO:0032682)	3/17	0.0035
Positive regulation of tumor necrosis factor superfamily cytokine production (GO:1903557)	5/81	0.0036
Response to interferon alpha (GO:0035455)	3/18	0.0039
Positive regulation of cell population proliferation (GO:0008284)	11/474	0.0044
Positive regulation of interferon alpha production (GO:0032727)	3/20	0.0050
Regulation of response to cytokine stimulus (GO:0060759)	3/22	0.0065
Response to cytokine (GO:0034097)	6/150	0.0071
Regulation of interferon gamma-mediated signaling pathway (GO:0060334)	3/23	0.0072
Regulation of interferon alpha production (GO:0032647)	3/25	0.0090
Apoptotic process (GO:0006915)	7/231	0.0114
Negative regulation of cytokine production (GO:0001818)	6/182	0.0151
Apoptotic mitochondrial changes (GO:0008637)	3/33	0.0161
Negative regulation of extrinsic apoptotic signaling pathway via death domain receptors (GO:1902042)	3/34	0.0174
Positive regulation of cytokine production (GO:0001819)	8/335	0.0181
Cellular response to tumor necrosis factor (GO:0071356)	6/194	0.0192
Response to interferon gamma (GO:0034341)	4/80	0.0210
Positive regulation of intrinsic apoptotic signaling pathway (GO:2001244)	3/40	0.0237
Regulation of chemokine production (GO:0032642)	3/42	0.0262
Regulation of extrinsic apoptotic signaling pathway via death domain receptors (GO:1902041)	3/42	0.0262
Regulation of type I interferon production (GO:0032479)	4/89	0.0269
Regulation of cytokine production (GO:0001817)	5/150	0.0283
Regulation of endothelial cell apoptotic process (GO:2000351)	3/44	0.0284
Regulation of response to interferon gamma (GO:0060330)	2/14	0.0330
Positive regulation of defense response (GO:0031349)	4/98	0.0331
Cellular response to cytokine stimulus (GO:0071345)	9/482	0.0341
Positive regulation of response to cytokine stimulus (GO:0060760)	2/15	0.0356
Regulation of ERK1 and ERK2 cascade (GO:0070372)	6/238	0.0368
Regulation of intrinsic apoptotic signaling pathway (GO:2001242)	3/52	0.0374
Positive regulation of endothelial cell apoptotic process (GO:2000353)	2/16	0.0374
Positive regulation of myeloid leukocyte cytokine production involved in immune response (GO:0061081)	2/16	0.0374
Negative regulation of gene expression (GO:0010629)	7/322	0.0374
Regulation of cell migration (GO:0030334)	8/408	0.0375
Regulation of interleukin-6 production (GO:0032675)	4/110	0.0428
Positive regulation of interferon gamma production (GO:0032729)	3/57	0.0450
Negative regulation of immune system process (GO:0002683)	2/19	0.0485
Tumor necrosis factor-mediated signaling pathway (GO:0033209)	4/116	0.0486
Positive regulation of cellular process (GO:0048522)	10/625	0.0494
**KEGG**		
Autoimmune thyroid disease	6/53	1.33 × 10^−5^
Cell adhesion molecules	7/148	4.01 × 10^−4^
ABC transporters	4/45	0.0014
IL-17 signaling pathway	5/94	0.0021
Necroptosis	5/159	0.0203
Endocytosis	6/252	0.0309

Pathway enrichment analysis of DRGs in LEC + MCF-7 spheroids transfected with miR193a and the mimic control. Analysis was performed by comparing the BioPlanet, GO Biological Process, and the KEGG on the Enrichr website by uploading DRGs obtained by the Transcriptome Analysis Console (TAC). The table lists the number of regulated genes compared to the total number of genes in the pathway (second column) and the *p*-value adjusted for multiple testing (last column).

**Table 4:** List of common genes involved in the enriched pathways.

Gene Symbol	Description	Log2 FC	FDR *p*-Value
IFIT1	interferon-induced protein with tetratricopeptide repeats 1	−4.29	5.98 × 10^−8^
OAS1	2-5-oligoadenylate synthetase 1	−3.2	2.65 × 10^−6^
OAS2	2-5-oligoadenylate synthetase 2	−2.53	2.65 × 10^−6^
HLA-A	major histocompatibility complex, class I, A	−2.51	3.08 × 10^−7^
IRF9	interferon regulatory factor 9	−2.35	3.08× 10^-7^
IFIT3	interferon-induced protein with tetratricopeptide repeats 3	−2.01	1.38 × 10^−5^
IFIH1	interferon-induced, with helicase C domain 1	−2	2 × 10^−4^
B2M	beta-2-microglobulin	−1.99	3.37 × 10^-5^
DDX58	DEAD (Asp-Glu-Ala-Asp) box polypeptide 58	−1.74	2 × 10^−4^
USP18	ubiquitin-specific peptidase 18	−1.65	6 × 10^−4^
OAS3	2-5-oligoadenylate synthetase 3	−1.58	9 × 10^−4^
HLA-F	major histocompatibility complex, class I, F	−1.56	2.83 × 10^−6^
PARP14	poly(ADP-ribose) polymerase family member 14	−1.55	1.21 × 10^−5^
HLA-DRA	major histocompatibility complex, class II, DR alpha	−1.53	2.1 × 10^−3^
SAMHD1	SAM domain and HD domain 1	−1.5	9.46 × 10^−5^
BTN3A2	butyrophilin, subfamily 3, member A2	−1.38	9.7 × 10^−3^
HERC5	HECT and RLD domain containing E3 ubiquitin protein ligase 5	−1.29	0.0483
CFB	complement factor B	−1.15	0.0178
EIF2AK2	eukaryotic translation initiation factor 2-alpha kinase 2	−1.11	1.9 × 10^-3^
OASL	2-5-oligoadenylate synthetase-like	−1.09	0.0204
TAP2	transporter 2, ATP-binding cassette, sub-family B (MDR/TAP)	−1.09	0.0125
ABCC3	ATP-binding cassette subfamily C member 3	0.93	0.046
ZFP36	ZFP36 ring finger protein	1.14	0.0278
ABCC2	ATP-binding cassette subfamily C member 2	1.33	8 × 10^-4^
CLDN3	claudin 3	1.42	4.2 × 10^-3^
MMP13	matrix metallopeptidase 13	1.93	5.61 × 10^−6^
S100A7	S100 calcium-binding protein A7	2.73	3.08 × 10^−7^
MMP1	matrix metallopeptidase 1	3.06	6.61 × 10^−8^

DRGs in LEC + MCF-7 spheroids transfected with miR193a and the mimic control implicated in the enriched pathways. The table lists the common genes involved in the significant enriched pathways from BioPlanet, GO Biological Process, and the KEGG by uploading DRGs obtained by the Transcriptome Analysis Console (TAC). Fold changes (FC) and adjusted *p*-values (FDR *p*-value) are depicted in the third and fourth columns, respectively.

**Table 5:** List of apoptosis-associated genes regulated by miR193a-3p in spheroids.

Gene Symbol	Description	Log2 FC	FDR *p*-Value
IFI6	interferon, alpha-inducible protein 6	−4.84	9.24 × 10^−8^
PSMB9	proteasome subunit beta 9	−2.95	8.75 × 10^−7^
HLA-B	major histocompatibility complex, class I, B	−2.78	9.24 × 10^−8^
IFITM1	interferon induced transmembrane protein 1	−2.44	01.58 × 10^−7^
PSMB8	proteasome subunit beta 8	−2.41	2.52 × 10^−6^
IFI27	interferon, alpha-inducible protein 27	−2.35	1.2 × 10^−3^
HLA-C	major histocompatibility complex, class I, C	−1.97	8.86 × 10^−5^
STAT1	signal transducer and activator of transcription 1	−1.94	3.28 × 10^−6^
RSAD2	radical S-adenosyl methionine domain containing 2	−1.56	0.0235
CCL5	chemokine (C-C motif) ligand 5	−1.55	4 × 10^−4^
IFIT2	interferon-induced protein with tetratricopeptide repeats 2	−1.42	2 × 10^−4^
HLA-G	major histocompatibility complex, class I, G	−1.39	2 × 10^−4^
UBE2L6	ubiquitin-conjugating enzyme E2L 6	−1.39	6.2 × 10^−3^
ESR1	estrogen receptor 1	−1.38	0.0371
TAP1	transporter 1, ATP-binding cassette, sub-family B (MDR/TAP)	−1.34	1.2 × 10^−3^
IFITM3	interferon induced transmembrane protein 3	−1.24	8.84 × 10^−5^
BCL2	B-cell CLL/lymphoma 2	−1.18	9.4 × 10^−3^
PLSCR1	phospholipid scramblase 1	−1	4.6 × 10^−3^
TNFSF10	tumor necrosis factor (ligand) superfamily, member 10	−0.9	0.024
HPCAL1	hippocalcin-like 1	0.85	0.0129
TNFRSF12A	tumor necrosis factor receptor superfamily, member 12A	0.87	0.0216
S100A6	S100 calcium-binding protein A6	0.9	4.7 × 10^−3^
FLNA	filamin A, alpha	0.91	0.0137
AHR	aryl hydrocarbon receptor	1.05	0.024
DUSP6	dual specificity phosphatase 6	1.09	0.0121
NR4A2	nuclear receptor subfamily 4, group A, member 2	1.1	1.9 × 10^−3^
FGB	fibrinogen beta chain	1.13	6 × 10^−4^
MT1X	metallothionein 1X	1.21	0.0102
PHLDA2	pleckstrin homology-like domain, family A, member 2	1.22	6 × 10^−4^
AKR1C3	aldo-keto reductase family 1, member C3	1.33	3.1 × 10^−3^
RGS5	regulator of G-protein signaling 5	1.44	3 × 10^−4^
LGALS1	lectin, galactoside-binding, soluble, 1	1.48	1.9 × 10^−3^
S100A9	S100 calcium-binding protein A9	1.49	4 × 10^−4^
HMOX1	heme oxygenase 1	1.55	1.6 × 10^−3^
AREG	amphiregulin	1.61	6.5 × 10^−3^
S100A8	S100 calcium-binding protein A8	2.26	2.83 × 10^−6^

DRGs in LEC + MCF-7 spheroids transfected with miR193a and the mimic control implicated in the enriched pathways. The table lists the genes involved in the apoptotic pathways from BioPlanet, GO Biological Process, and the KEGG. Fold changes (FC) and adjusted values (FDR *p*-value) are depicted in the third and fourth columns, respectively.

## Data Availability

All data supporting the findings of this study are available within the article and its supplemental data file or from the corresponding author upon reasonable request.

## Supplementary Materials

The following are available online at https://www.mdpi.com/article/10.3390/cells12030389/s1. Table S1: Differentially regulated genes (DRGs) in miR193a-3p transfected spheroids. Table S2: Pathway enrichment analysis of DRGs in miR193a-3p transfected spheroids using Bioplanet, GO Biological Process and KEGG.
